# Dynamic Polaronic Control of Metal Cluster Adaptability on Reducible Oxides

**DOI:** 10.1021/jacs.5c13140

**Published:** 2026-02-03

**Authors:** Lulu Li, Julian Geiger, Pol Sanz Berman, Núria López

**Affiliations:** † Institute of Chemical Research of Catalonia (ICIQ-CERCA), The Barcelona Institute of Science and Technology (BIST), Tarragona 43007, Spain; ‡ Universitat Rovira i Virgili, Tarragona 43002, Spain

## Abstract

Metal–oxide interactions are ubiquitous in many technological applications and involve a complex interplay between the oxide support and the metal nanoparticle. Particularly, it has been proposed that in strong metal–support interaction, the defect chemistry affects the metal cluster morphology. Here we develop a physics-guided machine learning framework to decode these interactions using Pt_7_ and Pt_13_ representative of planar and tridimensional clusters, analyzing the impact of across oxygen vacancy concentrations of CeO_2–*x*_ = 0–12.5% (528 configurations). Our models (*R* ^2^ > 0.97) reveal that polaron swarms, rather than defect concentrations, predominantly control cluster shape and charge through size-dependent pathways. The framework yields quantitative design principles for defect-driven catalyst optimization and provides a general methodology for systematic mechanisms of metal–support interactions across diverse catalyst systems.

## Graphical abstract

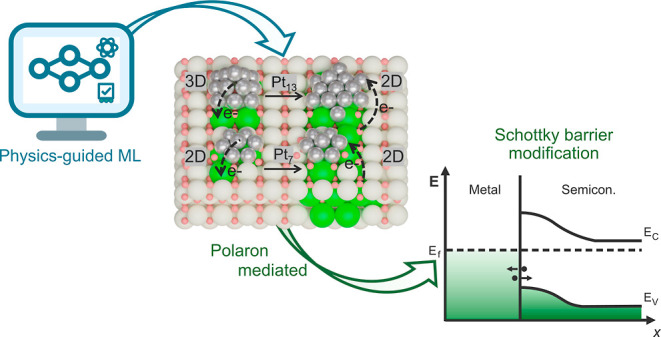

## Introduction

Metal oxide-supported catalysts occupy a central position in many technologies, particularly in heterogeneous catalysis, where metal–support interactions (MSIs) and electronic metal–support interactions (EMSIs) play a decisive role in determining catalyst activity, selectivity, and durability.^1–4^ Already in the 1980s, Tauster and colleagues suggested that the Schottky barrier created at the metal-oxide interface could affect metal cluster morphology^5,6^ but the atomistic mechanism of how this shape change occurs remains unknown.

Among metal-oxide catalytic systems, platinum clusters supported on ceria (Pt/CeO_2_) demonstrate superior performance in essential reactions, including CO oxidation,^7,8^ the water–gas shift reaction,^9,10^ and hydrogenation processes.^11,12^ This remarkable catalytic activity arises from the unique properties of ceria (CeO_2_), especially its ability to stabilize atomically dispersed or nanoclustered Pt species, facilitate rapid redox cycling, and promote efficient oxygen activation.^13–15^

The rich defect chemistry of ceria is central to its function.^16,17^ Thus, the concentration and distribution of oxygen vacancies (O_v_) and the accompanying formation of localized Ce^3+^ polarons states impact the reducibility of surface sites.^18–22^ Importantly, these defects are not static, O_v_ forms and migrates both on the surface and within the bulk under reaction conditions, with activation barriers ranging from 0.40 to 0.52 eV.^23,24^ Moreover, polarons associated with these defects exhibit faster hopping and redistribution within 0.25 eV.^25–27^ At high defect densities, the interplay between charge carriers and structural defects becomes increasingly intricate.^16,28^ While previous works have identified descriptors such as vacancy formation energy and redox capacity as key factors in ceria-based catalytic behavior,^29,30^ these descriptors primarily address static structures and may not fully account for how subtle changes in defect populations dynamically modulate polaron distribution, oxygen mobility, and ultimately catalytic performance.^31–33^

On these dynamic ceria substrates, supported Pt clusters exhibit their own structural fluxionality, continuously sampling a spectrum of atomic configurations controlled by both thermodynamics and kinetics.^34^ This inherent fluxionality makes them highly sensitive to the local environment, undergoing support-mediated transformations and displaying memory-dictated dynamics.^9,35,36^ Cluster size emerges as a critical parameter, modulating structural adaptability across different size regimes and consequently yielding diverse and tunable catalytic properties.^37–41^ Notably, supported Pt clusters on ceria transition from 2D planar to 3D compact around Pt_7_–Pt_8_, making a key size-dependent structural switch.^42,43^

Furthermore, the dynamics of ceria defects and Pt clusters are not isolated phenomena, both O_v_ formation and EMSIs-driven charge transfer^44^ can independently generate polarons, which strongly repel each other. Although previous studies have quantitatively explored electron redistribution at Pt/Ceria interfaces,^45,46^ the interplay of polarons from different origins, as well as their strong mutual exclusion and dynamic competition at the interface, still requires systematic exploration. This dynamic polaron–polaron repulsion, combined with the fluxional nature of supported clusters, produces an extremely complex geometric and electronic structure entanglement. The resulting catalytic system is governed by nonlinear couplings among defect chemistry, atomic rearrangement, and electronic structure.

Unraveling these interplay effects is challenging for both traditional experimental and computational approaches. High-resolution experimental techniques cannot capture fast, spatially heterogeneous dynamics,^47^ while traditional computational approaches struggle to explore the rugged energy landscape created by processes such as vacancy migration, dynamic polaron distributions, and cluster reshaping.^48^ The vast configurational space defies simple structure–property correlations.^49^ Advances in machine learning (ML) offer promising alternatives,^50–52^ with physics-guided approaches successfully identifying hidden patterns while maintaining interpretability.^53–55^

In this work, we present a computational framework that integrates density functional theory (DFT) with ML methods to investigate polaron dynamics and Pt cluster morphology on CeO_2–*x*_. By comparing Pt_7_ and Pt_13_, we demonstrate that cluster size governs shape variability and couples strongly with ceria redox behavior, with cluster distortion and charge redistribution being closely entangled. Although the cooperative roles of cluster ensembles and charge–structure correlations are recognized in heterogeneous catalysis,^9,36,56,57^ we extend these concepts to the dynamic regime of oxygen-deficient ceria, providing quantitative evidence that polaronic states mediate cluster adaptability and interfacial coupling. Our findings underscore how defect chemistry and polaron dynamics collectively govern cluster behavior, providing a path for energy evaluation of complex systems.

## Results and Discussion

### Pt_cluster_ Adsorption on CeO_2–*x*_ Surfaces

Here, Pt_7_ and Pt_13_ were selected as representative two-dimensional (2D) and three-dimensional (3D) clusters. Pt_7_ are structurally stable and resist sintering, maintaining relatively rigid and well-defined configurations.^58^ In contrast, Pt_13_ clusters exhibit pronounced structural diversity, dynamically exploring multiple locally stable geometries.^59,60^ These two sizes were deliberately chosen because they mark the atomic-scale crossover between compact 3D and planar 2D geometries, which has been well established in the literature.

For each size, we first systematically investigated how shape dictates the degree of wetting and adsorption behaviors of Pt clusters on the pristine CeO_2_(100) surfaces by PBE + U approaches.^61^ Representative stable adsorption configurations were identified in Note S1, Figures S1 and S2. Each cluster size exhibits a distinct spectrum of stable shapes, ranging from compact motifs to flattened liquid-like configurations. To provide a quantitative geometric feature for the extent of cluster–support interaction, we introduced the concept of “contact degree”.^62^ This is analogous to wetting in macroscopic systems and thus defined as the percentage of Pt atoms in contact with the oxide surface. The values were indicated in Figure 1a, b. Pt_7_ structures evolve from moderately wetted two-layer (Pt_7__2l, 71%) to fully planar (Pt_7__flat, 100%), while Pt_13_ clusters access a broader range, including 3d (Pt_13__3d, 46%), hollow (Pt_13__bj, 62%), 2l (Pt_13__2l, 68%), rod (Pt_13__rod, 85%), and flat (Pt_13__flat, 100%).

**Figure 1:**
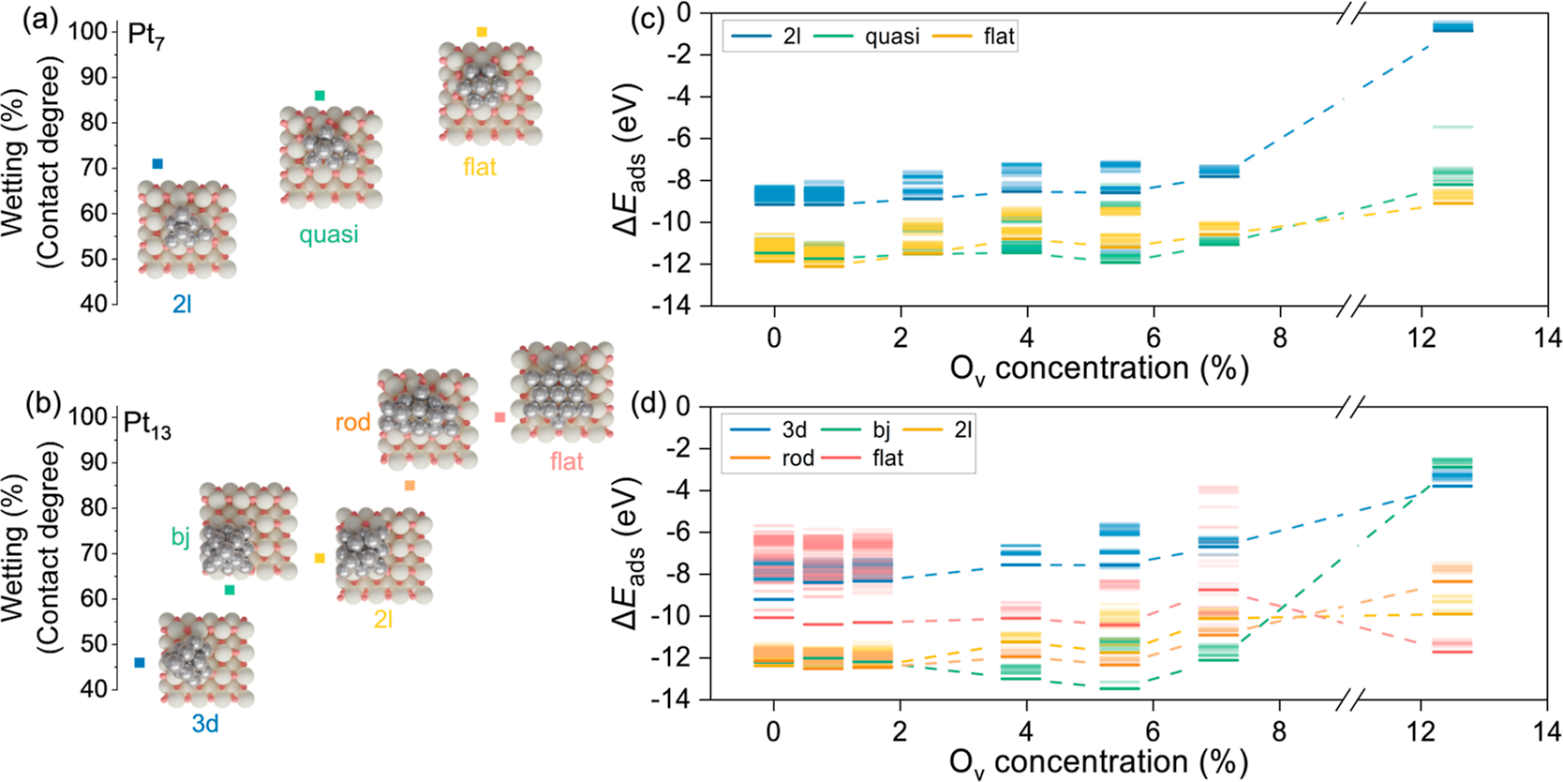
Influence of cluster geometry and CeO_2_ O_v_ concentration on Pt wetting and adsorption strength. (a,b) Wetting behavior (contact degree, %) of Pt_7_ (a) and Pt_13_ (b) clusters on CeO_2_ as a function of cluster geometry. Top-view snapshots show representative “2l” (blue), “quasi” (green) and “flat” (yellow) shapes for Pt_7_, and “3d” (blue), “bj” (green), “2l” (yellow), “rod” (orange) and “flat” (pink) shapes for Pt_13_. (c,d) Δ*E*_ads_ versus O_v_ concentration for each geometry of Pt_7_ (c) and Pt_13_ (d). Individual adsorption events are shown as horizontal ticks; dashed lines trace the most stable Δ*E*_ads_ for each geometry.

To further elucidate the role of ceria defects in supported cluster stability, we introduced O_v_ defects into the bulk of CeO_2_(100), in concentrations ranging from pristine (0%) to highly defective states (12.5%), remaining slightly below the upper nonstoichiometry limit of *x* ≈ 0.28 (i.e., 14% O_v_) for the fluorite CeO_2_ phase.^63,64^ The CeO_2_(100) surface, known to exhibit high reducibility and strong Pt anchoring behaviors, provides an ideal substrate for probing how defect chemistry modulates wetting and cluster stability.^65^ Detailed methods for bulk defect generation are described in Note S2. We also performed corresponding Pt_cluster_/CeO_2–*x*_(111) calculations, and the results (Figure S10) indicate that CeO_2_(111) is more inert than CeO_2_(100) (see Note S1).

Adsorption energies (Δ*E*_ads_) for Pt_7_ and Pt_13_ clusters across these O_v_ concentrations were evaluated compared to the gas phase (Figures 1c,d, S7 and S8). Generally, increasing O_v_ concentration within ceria bulk led to decreased cluster stability. A pronounced stability drop occurred at high defect concentrations (≥7.03%), while at lower to moderate O_v_ levels, adsorption energies remained relatively stable within a narrow window of ∼1.5 eV. For Pt_7_ clusters, the Pt_7__2l configuration was the least stable, whereas Pt_7__quasi and Pt_7__flat configurations exhibited similarly higher stability, suggesting possible interconversion under reaction conditions.

Pt_13_ clusters exhibited more diverse behavior. Most Pt_13_ configurations showed reduced stability with increasing O_v_ concentration in Figure 1d. However, the Pt_13__flat configuration exhibits a stability crossover at elevated O_v_ concentrations. While less favorable at low vacancy densities, Pt_13__flat becomes energetically competitive and even preferred once O_v_ exceeds approximately 7%. This result shows how bulk defects can dynamically reshape supported-cluster stability landscapes.

Despite size differences, Pt_7_ and Pt_13_ clusters exhibited similar Δ*E*_ads_ ranges, indicating that at the nanometre scale, cluster stability is more sensitive to wetting and interfacial interaction than to absolute cluster size. For both cluster sizes, liquid-like planar configurations were generally more stable than compact 3D structures. Nonetheless, intrinsic metal–oxide interactions, driven by interfacial charge transfer, impose limits on the extent of cluster spreading.^7^ This was particularly evident in larger clusters (Pt_13_), where compact configurations such as Pt_13__bj, Pt_13__rod, and Pt_13__2l structures dominated stability at CeO_2_(100) (Figure S8). Upon increasing O_v_ concentration, substantial surface reconstruction shifted stability toward more liquid-like Pt_13__flat, underscoring structural adaptability as a critical factor to maintain optimal metal–support interactions.

### Dynamic Behavior of Pt_cluster_/CeO_2–*x*_ Systems

Beyond the general stability trends and shape changes previously discussed, an essential characteristic of Pt_cluster_/CeO_2–*x*_ systems is their intrinsic electronic and structural dynamism. This is characterized by multiple energetically accessible states within a single cluster morphology under a specific O_v_ condition, as shown in Figure 1c,d. These energy variations are influenced by the density and distributions of polarons on the surface and throughout the system, which are dynamic, as demonstrated in Figures S7 and S8. As we mentioned before, this diverse nature originates primarily from intrinsic Pt/CeO_2_ interactions, which are inherently present even under pristine oxide conditions. Upon introduction of O_v_ defects, the polaronic behavior is amplified, with substantial spatial and energetic variability across different polaron sites, reflecting continuous evolution in the local electronic environment (Figures S18–S21). Pt_13_ clusters illustrate a clear example at an O_v_ concentration of 7.03%, where adsorption energies exhibit a wide variation of ∼8⁡eV , far exceeding the total energy change associated with the initial stages of defect formation. Consequently, rather than possessing a single well-defined adsorption energy, each nominally identical Pt cluster configuration corresponds to a diverse range of energetically accessible state.

Detailed polaron sampling further demonstrates structural and energetic fluctuations directly linked to dynamic electron localization processes. Both Pt_7_ and Pt_13_ clusters undergo notable shifts in stability and spontaneous structural reshaping driven explicitly by evolving polaron distributions. For instance, Pt_7__3d clusters transform into energetically favored more liquid-like configurations (quasi) at multiple O_v_ concentrations (0.78%, 3.91%, and 5.47%, Figure S7). These spontaneous structural transformations emphasize the direct coupling between electronic localization and structural fluxionality. The underlying mechanism of such structural adaptability is closely related to localized electronic restructuring at polaronic sites around O_v_. Charge and geometric distortion analyses explicitly reveal that increased electron localization correlates with elongated Pt–O bonds and modified Ce^3+^–Ce^3+^ interactions. The interplay of bulk oxygen vacancy distributions, dynamically varying polarons, and structural transformations creates massive complexity in Pt/CeO_2–*x*_ systems, which makes it difficult to only focus on identifying simple features or intuitive relationships to explore this behavior. This demonstrates that a static, single-structure picture is insufficient. Instead, one must treat the Pt/CeO_2–*x*_ system as a collection of electronic and geometric microstates (ensembles), each contributing to the observed material behavior.

### Features Organization

The complex Pt/CeO_2–*x*_ system was decomposed into three logical domains based on physical relevance: the Pt cluster, the Pt–O interface, and the CeO_2–*x*_ support (Figure 2a).

**Figure 2:**
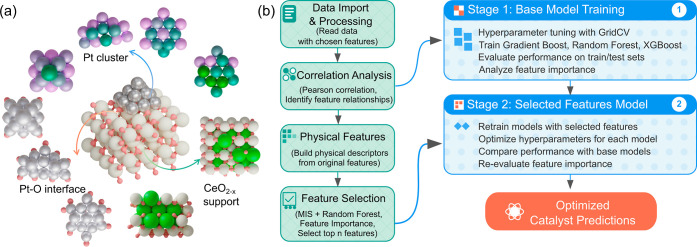
Features category and machine-learning workflow (a) Structural representation of the key components: Pt clusters (top), Pt–O interface region (bottom left), and CeO_2–*x*_ support (bottom right). (b) Workflow diagram of the ML pipeline showing data processing, correlation analysis, physical feature engineering, feature selection, and the two-stage model training process.

For the Pt cluster region, two key features were defined: the net electron transfer (Δ*q*, from Pt to CeO_2–*x*_) and the root-mean-square deviation (RMSD) relative to the gas-phase cluster of each size, quantifying electronic and geometric distortion, respectively. The Pt–O interface was characterized using six features, including Pt–O bond count, the total and statistical distribution of Pt–O bond distances (sum, mean, min, max, and standard deviation). These features capture the extent and nature of metal–support interactions. The CeO_2–*x*_ region was represented by 11 features covering: (i) defect and polaron-related characteristics such as O_v_ concentrations, surface and total polaron counts; (ii) surface strain indicators (e.g., number of surface O atoms near Ce^3+^); (iii) spatial statistics of Ce^3+^–Ce^3+^ distances (sum, mean, min, max, and standard deviation); and (iv) electrostatic energies including polaron–polaron repulsion and polaron–lattice interactions.^66^ These features were selected to capture fundamental physics governing the material behavior explicitly: the Pt cluster features directly quantify electronic-structural coupling within clusters, Pt–O interface metrics characterize interfacial anchoring strength critical for metal–support interactions, and CeO_2–*x*_ support features reflect the redox dynamics and polaronic effects inherently linked to catalytic activity (see Note S3, Table S1). To identify both redundant physically meaningful features, we computed the Pearson correlation matrix, shown in Figure S11, which quantifies (strength and direction) linear relationships among all structural and electronic features. In addition to the primary features, the derived features were constructed through physical transformations and interactions between sets, such as pairwise energy terms and local structural perturbation indices, which also conduct a Pearson correlation matrix in Figure S12.

### Interpretability of Machine-Learning Framework

To decode the complex factors and hidden relations of Pt cluster behavior on CeO_2–*x*_, we developed a modular ML workflow linking DFT-derived structural and electronic features to targeted variables (in this work, Δ*E*_ads_), as outlined in Figure 2b. The analysis was conducted in two stages. Stage 1 employed original features (Table S3) to train regression models, including Gradient Boosting (GB), Random Forest (RF), and eXtreme Gradient Boosting (XGBoost). In stage 2, composite features were derived through the combination of the top original features. Feature selection was guided by correlation analysis and clustering methods to ensure that the final set of features was both informative and nonredundant (see Note S4 for details). The dataset was randomly divided into training (85%) and test (15%) sets with shuffling and a fixed random seed to ensure reproducibility. Hyperparameter tuning for the three models is provided in Figures S13–S15. All feature selection, hyperparameter tuning, and model selection were conducted exclusively within the training set using a 5-fold cross-validation approach. Furthermore, to ensure statistical rigor, we applied Bonferroni-adjusted correlation analysis with a threshold of *p* < 0.05. This approach identifies statistically robust feature interactions, thereby revealing physically meaningful relationships that inform quantitative guidelines for Pt/CeO_2_ catalyst optimization. Detailed statistical procedures are provided in Note S4.

Three ML algorithms, GB, RF, and XGBoost, achieved accuracy with *R* ^2^ > 0.97 and RMSE < 0.41 eV (Figure 3a,c,e), validating that our physically motivated features capture the factors governing adsorption energetics across diverse cluster shapes and defect concentrations. Feature importance analysis, considering only features with importance scores exceeding 0.05, reveals consistent patterns across all models (Figure 3b,d,f). Interfacial features dominate the rankings, with Σ­(Pt–O dist) emerging as the primary predictor, contributing to 15–30%. This feature directly quantifies the cumulative metal–support anchoring strength. Notably, support-based features, namely Ce^3+^–Ce^3+^ distances and polaron count, consistently rank among the top four features, directly revealing that polaron distribution and interaction play a priority role in modulating the supported clusters.

**Figure 3:**
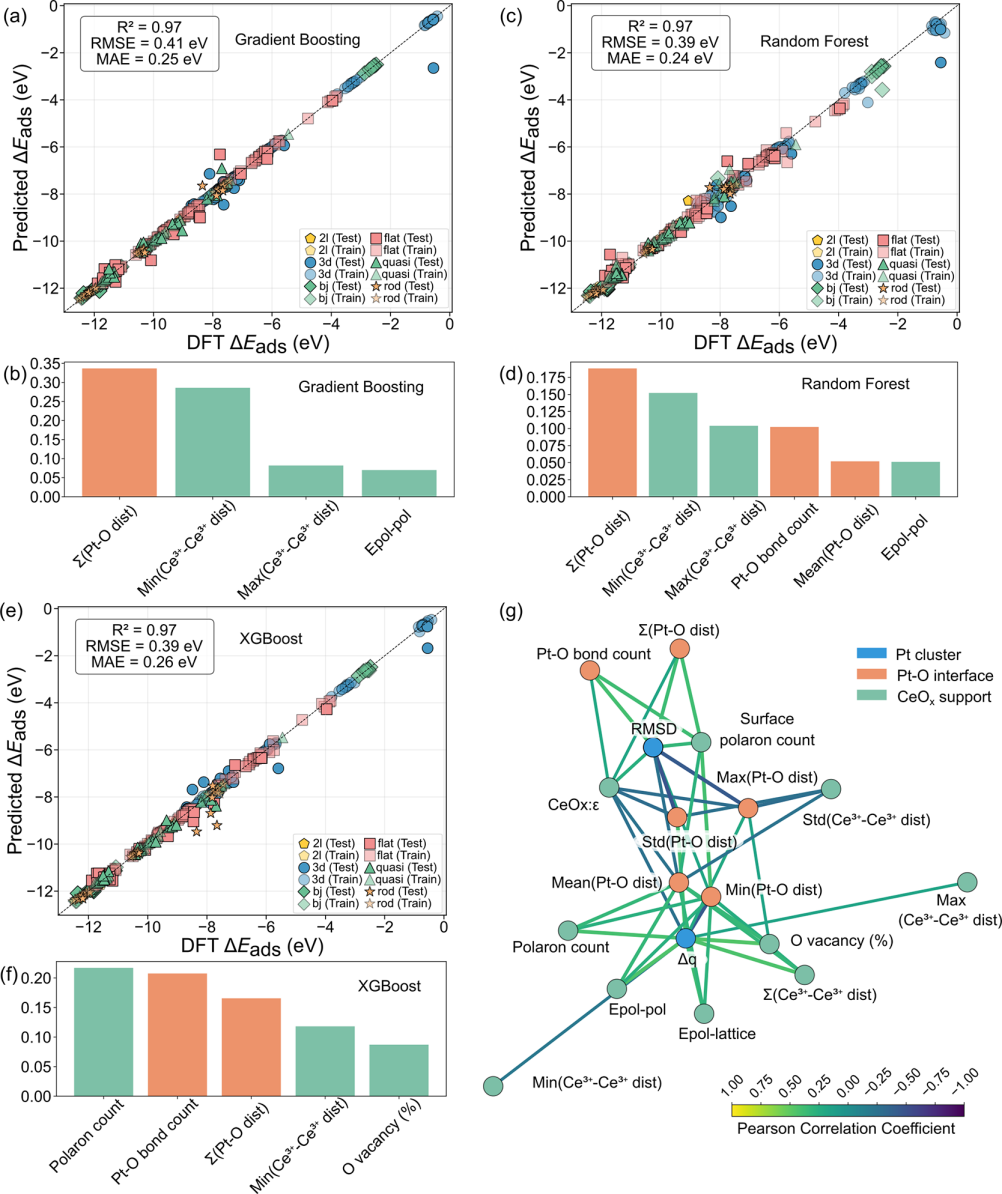
Model performance of Pt/CeO_2–*x*_ systems. (a,c,e) Parity plots comparing DFT-calculated adsorption energies with predictions from three ML models: GB (a) RF (c) and XGBoost (e). (b,d,f) Feature importance analysis for each model. (g) Correlation network of features. Edge colors and thickness represent the strength of the Pearson correlation.

Model-specific insights provide complementary perspectives. Both GB and RF emphasize Ce^3+^–Ce^3+^ distance statistics (min, max), revealing sensitivity to the extent of polaron interactions. XGBoost uniquely prioritizes polaron count as its most important feature, complementing the Ce^3+^–Ce^3+^ distance favored by other models. Together, these polaron-mediated features consistently dominated all models, providing robust evidence that polaronic effects control the adsorption energetics. In contrast, cluster-intrinsic properties (RMSD, Δ*q*) do not appear among top-ranked features, suggesting these represent outcomes rather than controlling factors.

To demonstrate generalizability and interpretability, we implemented a stage 2 feature engineering step, constructing physically motivated interaction terms, and validated this extension on the same dataset. As detailed in Figure S16, the primary value of stage 2 lies not merely in incremental predictive improvement (*R* ^2^ ≈ 0.98), but the feature importance analysis in stage 2 identifies top-ranked descriptors that closely align with those from stage 1, further enhancing interpretability and robustness. To assess model transferability, we tested the framework on larger Pt_19_ clusters on CeO_2_(100). The results (Figure S17) show that while direct extrapolation from the Pt_7_/Pt_13_ model yields limited accuracy, fine-tuning with a small portion of Pt_19_ data quickly restores the predictive power at a much lower computation cost. This emphasizes the importance of comprehensive yet efficient dataset preparation in achieving robust and generalizable ML performance for complex catalytic systems.

### Structure–Property Relationships of Pt_cluster_/CeO_2–*x*_ Systems

To explore the dependence of all physical features and search for the chemical rationale connecting support, cluster, and interface we analyzed a correlation network with Bonferroni-corrected correlations.^67^ As shown in Figure 3g, the network reveals a straightforward connection from the ceria support to the Pt cluster, which begins with support-level features, particularly the max and min Ce^3+^–Ce^3+^ distances, indicating that the polaronic interactions are the initial features. Then, these features exhibit strong correlations with other support and interface features, such as the number and length of Pt–O bonds, reflecting how oxide-driven electronic and structural perturbations shape the interface geometry. Only the central part of the network occurs with Pt cluster features, suggesting that variations in support and interface characteristics ultimately manifest in cluster-level structural and electronic responses. Furthermore, darker edge colors in the network indicate stronger correlations, which predominantly occur between interface and cluster descriptors, revealing the mediating role of the Pt–O interface in translating support-induced changes into cluster properties.

Figure 4 shows a flowchart based on the mechanism pathway using the ML-derived feature importance ranking and Bonferroni analysis. The ML performance demonstrates that Pt–O bond-distance, Ce^3+^–Ce^3+^ distances and polaron count are the most contributed features for structural response and adsorption stability. These findings are consistent with the correlation network analysis, highlighting the important roles of support-mediated electronic and interface features in determining cluster-level properties. The detailed mechanism can be summarized as follows: (i) oxygen vacancy creation and polaron generation in the ceria support create a dynamic Ce^3+^–Ce^3+^ swarm, (ii) these Ce^3+^–Ce^3+^ interactions effectively modulate the interface, i.e., Pt–O bond distances and counts, via the change of the interfacial electronic and structural properties, (iii) the interface further mediates the electronic and geometric responses of the Pt clusters, which are the active center in the Pt/CeO_2–*x*_ system.

**Figure 4:**
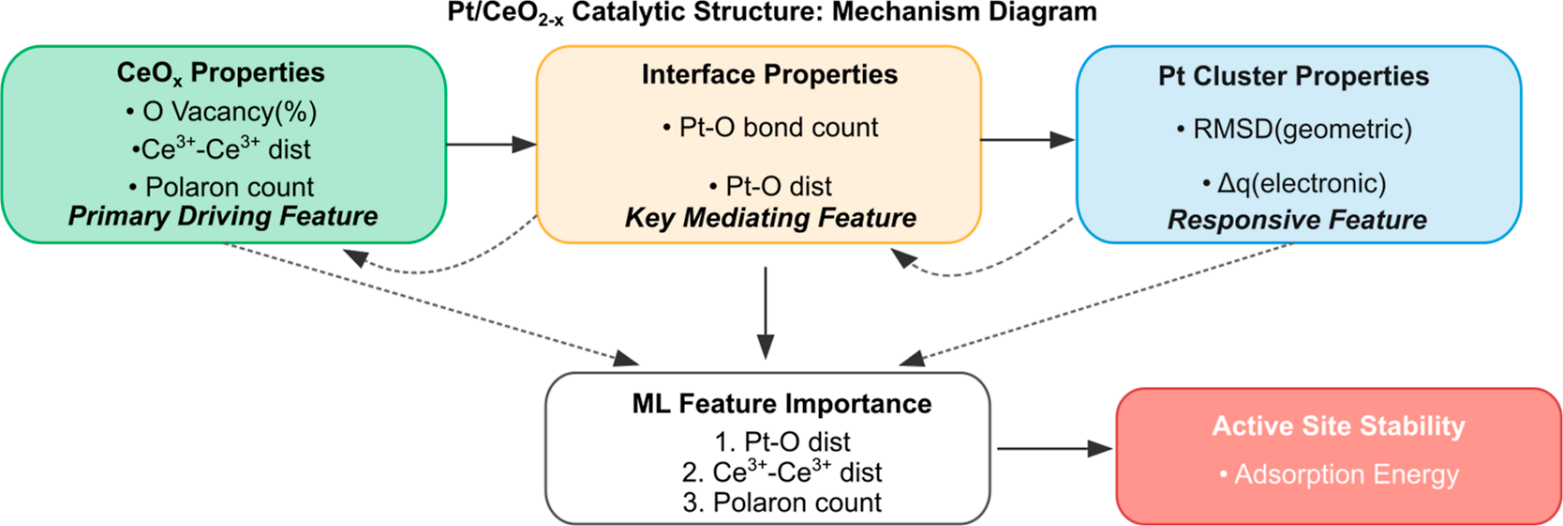
Mechanistic framework of Pt/CeO_2–*x*_ system. It highlights primary pathways and indirect interaction from oxide support properties to cluster properties.

The structural and electronic changes in Pt clusters, such as variations in size, charge state, and interfacial bonding, have been shown to significantly influence catalytic performance.^47,68^ *Operando* TEM studies revealed that increased fluxional behavior and O_v_ dynamics in the Pt/CeO_2–*x*_ system correlate directly with turnover frequency in CO oxidation.^36^ In this way, joining activity metrics with ML-informed structural features could yield a complete structure–reactivity function mapping, and in turn, allow for the design of metal cluster response to target performance.

### Geometric-Electronic Entanglement in Pt Clusters

Since Pt clusters are the main active species for further applications, Figure 5a,b present the probability density distributions for Pt_7_ (blue) and Pt_13_ (green) clusters in terms of Δ*E*_ads_, geometric feature (RMSD), and electronic feature (Δ*q*). Regarding binding strength, Pt_7_ clusters exhibit adsorption energies broadly distributed between approximately −12 to −6 eV, with a primary peak around −10 eV and a minor population near 0 eV. In comparison, Pt_13_ clusters exhibit adsorption energies ranging from approximately −14 to 0 eV, with two prominent peaks at –11 eV and −8 eV. Despite the size difference, both clusters show comparable adsorption energy ranges, suggesting that binding strength is not strongly dependent on cluster size. In contrast, their structural and electronic properties reveal a clear size-dependent behavior. For structural fluxionality, Pt_13_ clusters show pronounced distortion patterns, with RMSD values broadly distributed between 5.0 and 7.0 Å. Pt_7_ clusters, however, display a sharply peaked and narrow distribution centered around 3.8 Å, with minimal spread (3.6–4.0 Å), indicating more constrained geometries. The charge transfer behavior follows a similar trend. Pt_13_ clusters exhibit a wide range of Δ*q*, spanning from electron depletion (negative values) to electron accumulation (positive values), indicating dynamic and extensive charge redistribution between the cluster and support. In contrast, Pt_7_ clusters show a more limited range, with charge transfer consistently occurring from Pt to CeO_2–*x*_ (from ∼−0.8 |e^–^| to near 0), without a clear reversal in direction (bottom panel of Figure 5).

**Figure 5:**
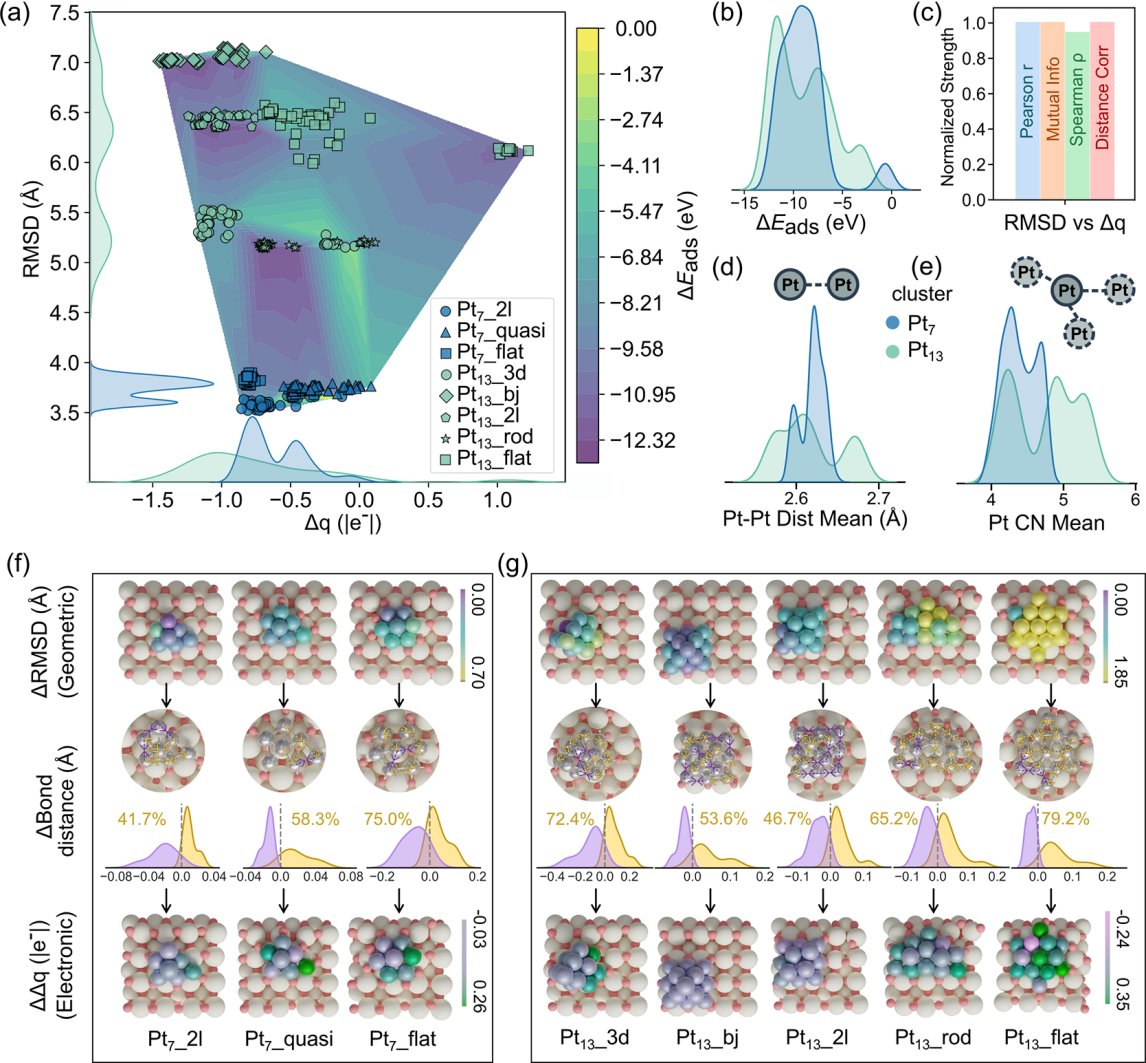
Structural–electronic coupling and behavior mapping of Pt clusters on CeO_2–*x*_. (a) Δ*q*–RMSD landscape colored by Δ*E*_ads_, overlaid with probability density distribution of RMSD and Δ*q* on cluters. (b) Probability density of Δ*E*_ads_. (c) Coupling strengths between RMSD and Δ*q* across four statistical metrics. (d,e) Probability density distributions of Pt–Pt bond distance mean and Pt coordination number (CN) mean. (f,g) Atomic-level changes from pristine to 12.5% O_v_ concentration for representative Pt_7_(f) and Pt_13_(g) cluster configurations. Top row: atomic distortion maps (ΔRMSD); middle row: Pt–Pt bond distance distribution (yellow indicates bond elongation, purple indicates bond contraction, arrows highlight only the significant bond distance variations); bottom row: charge redistribution maps (ΔΔ*q*).

In Figure 5c, the RMSD vs Δ*q* relationship exhibits strong coupling with normalized strengths approaching ∼0.9–1.0 for all four methods (Pearson *r*, Spearman ρ, mutual information, and distance correlation), demonstrating that structural distortion and charge transfer are fundamentally entangled. In contrast, the correlations involving adsorption energy are weaker and more method-dependent (Figure S22), suggesting that while both structural and electronic properties influence binding energy, these relationships are more complex and indirect compared to the direct RMSD-Δ*q* coupling. Therefore, geometric restructuring and charge redistribution in Pt clusters are inseparably linked, representing a unified structural-electronic response rather than independent phenomena. In addition to geometric (RMSD) and electronic (Δ*q*) features, Figure 5d,e incorporates two mechanistically interpretable structural characteristics: the average Pt–Pt bond distance and the average Pt coordination number (CN). Probability density reveals that Pt_13_ clusters show larger distributions in Pt–Pt bond distances (∼2.68 Å) and CN values (4.2–5.5), with respect to Pt_7_ (max bond distance, ∼2.62 Å; CN, 4.2–4.7). These distinctions further demonstrate the underlying microscopic basis of bond lengthening and undercoordinated Pt in distorted Pt clusters, providing a connection of factors that define the global Pt cluster behavior (RMSD, Δ*q*) and local atomic configuration, providing further details about the mechanistic background of Pt/CeO_2–*x*_ structural adaptiveness.

We also investigate the structure–property landscape of single configurations in Figure 5a. For compact (less wetting) configurations, such as Pt_7__2l, Pt_13__3d, and Pt_13__bj, they occupy the most constrained region, with limited distortion (ΔRMSD: Pt_7_: 0.1 Å; Pt_13_: 0.3 Å) and relatively narrow charge transfer windows (Pt_7_: from −0.8 to −0.4 |e^–^|; Pt_13_: from −1.2 to 0.0 |e^–^|), regardless of cluster size. These compact shapes are intrinsically stable and survive under different concentrations of defects and structural perturbations. In contrast, liquid-like shapes cover a larger structure and electron space. These structures have distorted geometries (ΔRMSD: Pt_7_: 0.3 Å; Pt_13_: 0.6 Å) as well as large charge redistribution between isolated fragments (Pt_7_: −0.8 to 0.0 |e^–^|; Pt_13_: −0.6 to 1.2 |e^–^|). Besides, it seems that the liquid-like Pt_13__flat structure has the widest property space, leading to a crossover in stability trends as observed in Figure 1d. While the intermediate morphologies exhibit moderate degrees of fluxionality. Pt_13__2l structures cover RMSD with 6.3–6.5 Å and charge transfer from −1.2 to −0.8 |e^–^|, which can be considered as an intermediate regime between rigid and adaptable.

Overall, such behaviors reveal that different Pt cluster sizes and configurations employ distinct stabilization strategies to maintain stability under varying redox environments. Pt_7_ clusters are confined within reduced structural freedom (primarily 2l with 71% wetting). Pt_13_ clusters represent a dual-pathway optimization, while all configurations fall within a comparable Δ*E*_ads_ range (approximately −14 to closely 0 eV), their underlying responses differ, that is, compact shapes (3d, bj) show less structural distortion and rely primarily on limited electronic modulation, whereas liquid-like configurations (flat) exhibit large geometric reorganization and concurrent charge redistribution. This reflects a compensation mechanism at the stability level; that is, when geometric fluxionality is constrained, electronic degrees of freedom are more actively engaged to stabilize binding, and vice versa. Such size and configuration-dependent trade-off provides a form of synergistic tuning,^69^ enabling Pt clusters to retain catalytic functionality via alternative structural or electronic pathways.^70^

### Atomic-Level Mechanistic Understanding of Entanglement

To obtain a more profound understanding of the correlations between geometric distortion and electronic redistribution within Pt clusters, we performed detailed atom-resolved analysis. Figure 5f,g depicts all the Pt cluster configurations under pristine and the highest defective case (12.5% O_v_), and a full set of configurations at varying defect concentrations can be found in Figures S23–S26, S30 and S31.

In Figure 5f,g, the distortion maps (ΔRMSD, top row) reveal that atomic displacements are most pronounced at specific interfacial sites. For Pt_7_, the maximum distortion reaches 0.70 Å, while for Pt_13_, one Pt atom exhibits a displacement as large as 1.85 Å. Compared with Pt_13_, Pt_7_ clusters exhibit more localized distortions, whereas Pt_13_ displays broader structural perturbations extending into the cluster interior. In both Pt_13_ configurations, several atoms show ΔRMSD values exceeding 1.5 Å, underscoring their enhanced structural fluxionality (yellow-colored atoms in Pt_13__rod and Pt_13__flat). The bond distance analysis (middle row) reveals clear patterns of bond elongation and contraction upon CeO_2_ reduction. Most interfacial Pt–Pt bonds tend to elongate. Notably, the interfacial Pt–Pt bond distances increase by 0.11 Å in Pt_7__flat and 0.17 Å in Pt_13__flat. These results indicate that defect-induced structural reorganization initiates at the Pt/CeO_2–*x*_ interface and propagates inward with configuration-dependency. The charge redistribution maps (ΔΔ*q*, bottom row) show corresponding electronic responses, that is, interfacial Pt atoms undergo more pronounced geometric distortion (up to 1.85 Å in Pt_13__flat) coupled with substantial electron accumulation (green regions, up to 0.35 |*e* ^–^| on Pt_13__flat). In contrast, interior atoms with minimal displacements (e.g., 0.251 Å in Pt_7__flat) still exhibit moderate charge transfer (∼0.02 |*e* ^–^|), further confirming the strong coupling between local structural distortion and charge redistribution at the atomic level.

Critically, this electron redistribution indicates a reversal of conventional electron transfer direction, from typical Pt → CeO_2_ direction in pristine systems to CeO_2–*x*_ → Pt back-donation under reduced conditions. This occurs through Ce^3+^ polaron formation and accumulation near cluster boundaries, when sufficient oxygen vacancies create localized Ce^3+^ states proximate to Pt clusters, these polaronic states mediate electron back-donation that inverts the charge transfer direction.^35,71^ Quantitatively, the onset of this charge-transfer reversal emerges when the O_*v*_ concentration exceeds approximately 7%, as revealed by atom-resolved charge variation analyses (Figures S23–S26). This reversal in charge transfer is expected to have significant implications for catalytic performance such as CO oxidation, where the valence state of Pt active sites is known to regulate CO adsorption strength (Δ*E*_CO_).^35^ As we calculated in Figure S32, the Δ*E*_CO_ decreases with increasing electron density on Pt, indicating that polaron-mediated back-donation weakens Pt–CO binding and could mitigate CO poisoning in catalytic processes. Furthermore, the projected density of states (PDOS) analysis (Figures S27–S29) corroborates this mechanism, showing enhanced Ce 4f electron density close to 0 eV and concurrent downshifts in the Pt d-band, confirming electron enrichment of Pt atoms through polaron-mediated charge transfer. It modulates the interfacial electronic alignment, which can be conceptually analogous to Schottky barrier modification in extended metal–semiconductor junctions. Such electronic redistribution modifies the Schottky barrier at the Pt/CeO_2–*x*_ interface, and provides direct evidence of defect-driven barrier modulation beyond traditional static description and shape change induction as early suggested.^5^

As illustrated in Scheme 1, oxygen vacancy formation creates dynamic Ce^3+^ states that propagate through the oxide network (left), modulate Pt–O interfaces (center), and control cluster electronic-geometric coupling (right). The strong structure-charge entanglement enables predictive catalyst design through physics-guided machine learning, establishing ceria as an active electronic modulator rather than a passive support.

**Scheme 1:**
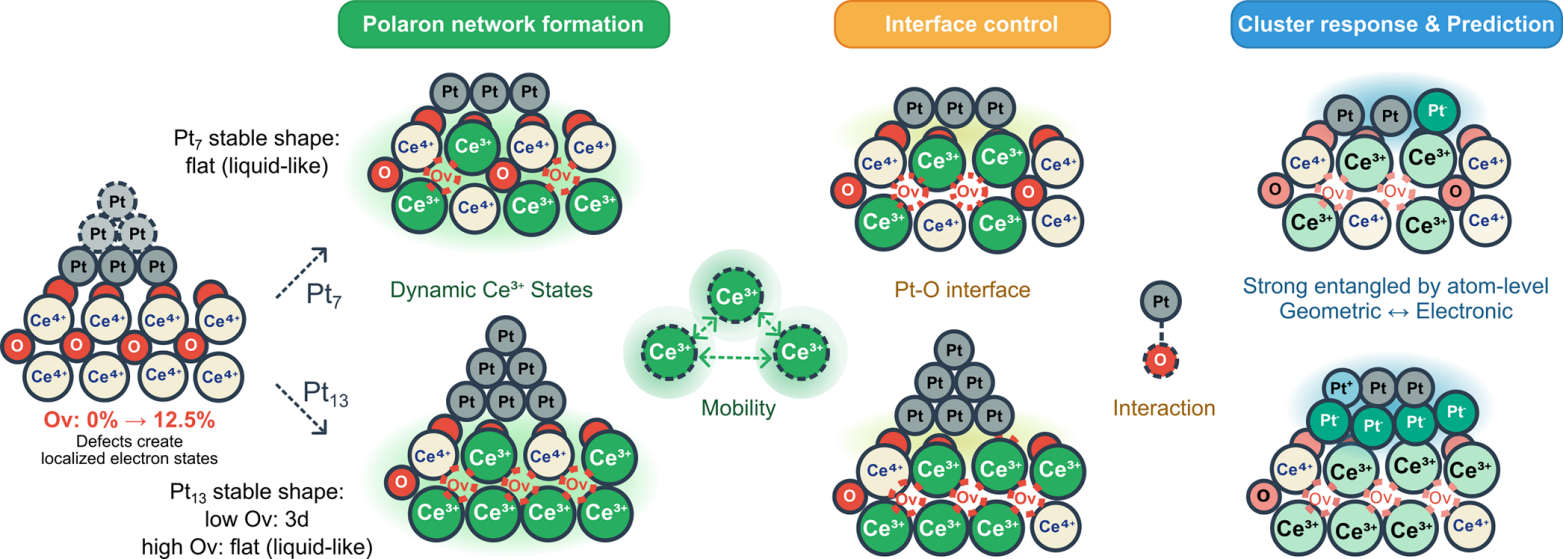
Polaron-Mediated Control of Metal Cluster Catalysts

## Conclusions

This work establishes a quantitative framework for understanding metal-oxide catalyst behavior through polaron-mediated control. By systematically investigating Pt_7_ and Pt_13_ clusters across varying O_v_ concentrations of ceria in Scheme 1, we demonstrate that traditional views of ceria as an oxygen reservoir are indirect, instead, ceria should be rather considered as an active electronic modulator with the help of polaronic states, taking the dynamic role of steering the process.

We discovered that although ceria defects appear as apparent variables, the fundamental controlling mechanisms operate through dynamic swarms of polaronic networks, creating spatially heterogeneous electronic landscapes (Scheme 1). This polaron-mediated control propagates from polaron network formation through interface control to cluster response: support-level Ce^3+^-Ce^3+^ interactions modulate Pt–O coordination environments, which subsequently govern cluster geometric and electronic properties with strong entanglement (*r* = 0.92). These insights provide an atomistic mechanistic explanation for the proposed shape changes of clusters suggested by Tauster and co-workers^5,6^ and expand upon classical Schottky barrier theory, demonstrating how nanoscale cluster and defect-induced polarons dynamically tune interfacial barrier heights through local states redistribution and interface electronic structure modification. In this way, our findings transform the traditional static picture of metal-oxide interactions into a rugged landscape of closely lying metastable minima and broaden the applicability of dynamically modulated Schottky barriers in catalyst design. This dynamic picture resonates with recent experimental observations on Pt/TiO_2_,^72^ reinforcing the emerging view that metal–oxide interfaces behave as adaptive, nonstatic entities. Our findings call for experimental validation through advanced microscopy (via shape changes) or spectroscopic techniques capable of tracking polaron dynamics and cluster restructuring simultaneously.

The identified size-dependent optimization pathways could transform strategies of catalyst design. It is also found that with an increasing number of stable active sites, Pt_7_ clusters can achieve the optimum constrained fluxionality by electronic modulation with the intact structure. Pt_13_ clusters are more flexible via the combination of the dual paths of electronic and geometric sensitivities. Importantly, the charge transfer reversal (CeO_2–*x*_ → Pt) at high defect concentration allows designing the synthesis of electron-rich Pt sites that are directly relevant for reactions that require enhanced back-bonding sites to increase catalytic performance.

Finally, the physics-guided ML workflow enacts actionable design principles: prioritise Ce^3+^-Ce^3+^ interactions over simple O_v_ concentration control, leverage the Pt–O coordination environment as the key control lever via polaron positioning, exploit the crossover behavior in Pt_13_ systems where compact to liquid-like transitions occur at high O_v_ levels, and cluster size is dictated by whether electronic tunability (Pt_7_) or geometric fluxionality (Pt_13_) is prioritized. Moreover, recent observations of reversible 3D to 2D transitions in ∼1.8⁡nm Pt/Al_2_O_3_ indicate that the structure-charge interplay uncovered here extends to the nanometer region, bridging the continuum from clusters to supported nanoparticles.^73^

Although our computational strategy offers previously unavailable detail on the mechanistic aspects, spectroscopy studies that can monitor polaron dynamics are needed for validation. Furthermore, we propose our dataset as a benchmark for future machine-learned interatomic potentials (MLIPs) for training in metal/oxide interfaces. The comprehensive geometric and electronic dataset generated in this work provides a useful training resource and rigorous benchmark for developing advanced MLIPs. While the current MLIPs training primarily rely on energy and force information there is an urgent need to incorporate more electronic and structural descriptors to predict complex structures behaviors and the corresponding applications accurately. Our rigorous approach can be extended to other metal-oxides, moving from an empirical screening to a predictive design based on physics-based guidelines.

## Methods

### Model Setup

In this study, the CeO_2_(100) surface was selected as a support for Pt_7_ and Pt_13_ clusters.^74^ Although the CeO_2_(111) facet is more stable than the (100), the latter can be directly synthesized and it is responsible for long-time trapping of single atoms at steps. (100)-like motifs are frequently found at step edges and corners of ceria nanoparticles, making this surface representative of catalytically active sites.^75,76^ In addition, this facet shows high reducibility, which promotes oxygen vacancy formation and strong electronic coupling with supported metals. The slab model for the CeO_2_(100) surface was constructed as (4 × 4) based on optimized ceria bulk. Pt clusters exhibited specific geometric shapes, following static structural optimization (Figures S1 and S2). To explore defect engineering strategies, O_v_ concentrations ranging from 0 to 12.5% were introduced into CeO_2_(100) through two distinct approaches. The first approach is used by generating O_v_ at various depths within the bulk layers, and the second method is removing O_v_ in the bulk region adjacent to the surface (Figure S3).

### Density Functional Theory

DFT calculations were conducted using the Vienna Ab initio Simulation Package (VASP).^77^ Electron–ion interactions were modeled through the projector augmented wave (PAW) method,^78^ and exchange–correlation effects were described using the generalized gradient approximation (GGA) with the Perdew–Burke–Ernzerhof (PBE) functional.^79^ A plane-wave cutoff energy of 500 eV was selected to achieve convergence of the total energies. All structural optimizations proceeded until the residual forces acting on each atom were below 0.015 eV/Å. Spin polarization was consistently included across all calculations to appropriately describe the magnetic moments characteristic of Ce^3+^ polarons. Due to the large size of CeO_2_(100) surface supercells, Brillouin zone sampling was restricted to the gamma point. Convergence criteria for electronic self-consistency were set stringently at 10^–5^ eV. To capture the localized nature of Ce 4f electrons and their strong correlation effects, we applied the DFT + U methodology proposed by Dudarev et al.,^80^ setting the effective Hubbard parameter (*U*_eff_ = *U* – *J*) to 4.5 eV for the Ce 4f orbitals.^81^ This treatment has been validated to reproduce accurately the formation and localization of Ce^3+^ states upon the introduction of oxygen vacancies.^13^

### Polaron Sampling

To explore the polaron distribution, we implemented a two-step geometric sampling strategy. Pt/CeO_2–*x*_ reveals two primary origins for the formation of Ce^3+^ sites: electron transfer between the Pt cluster and the support, and the introduction of oxygen vacancies (O_v_). Given our focus on the influence of polaron distribution, especially at potentially catalytic sites, in the first step each surface Ce atom was individually initialized in a reduced state to ensure the appearance of surface Ce^3+^ and to sample all possible localization sites. In the second step, the systems were fully relaxed under spin polarization, allowing the excess electrons to self-localize and redistribute freely across the entire model. This approach leads to spontaneous reorganization of polarons and final redistribution beyond the initial setup. No constraints were imposed on the relative positions of polarons during relaxation, and no energetic bias was introduced. Ce^3+^ site assignments were based on the localized magnetic moments of cerium atoms, using a threshold of 0.8 μ_B_, consistent with prior work.^44,82^ It should be emphasized that for configurations with identical final polaron distributions, the most stable structure was selected for further detailed analysis.

## Supplementary Material



## Data Availability

Simulated structures are available in the ioChem-BD database.^83^ (http://dx.doi.org/10.19061/iochem-bd-1-416).

## Notes

Additional computational details; defects construction; adsorption energies; polaron localization analysis; machine learning framework; atom-resolved information distribution, additional figures and tables (PDF)

## Notes

Code Availability: Code and raw data can be retrieved from our GitHub repository (https://github.com/LopezGroup-ICIQ/oxide_analysis_general).
