# Supplementary material for: Dynamic Polaronic Control of Metal Cluster Adaptability on Reducible Oxides
Source: J Am Chem Soc. 2026 Feb 3;148(12):12597–608. doi: 10.1021/jacs.5c13140 (PMC13047685; doi:10.1021/jacs.5c13140)
Supplement: Supplementary file 1 [file ja5c13140_si_001.pdf]

Supporting Information for Dynamic polaronic control of metal cluster adaptability on reducible oxides

Lulu Li,*,† Julian Geiger,† Pol Sanz Berman,† and Núria López*,†,‡

†*Institute of Chemical Research of Catalonia (ICIQ-CERCA), The Barcelona Institute of Science and Technology (BIST), Tarragona, 43007, Spain*

‡*Universitat Rovira i Virgili, Tarragona, 43002, Spain*

E-mail: lli@iciq.es; nlopez@iciq.es

Contents

Supporting Note 1 . . . . S4

Supporting Note 2 . . . . S6

Supporting Note 3 . . . . S7

Supporting Note 4 . . . . S8

Supporting Note 5 . . . . S10

List of Figures

S1 Adsorption configurations of Pt_7 clusters S12

S2 Adsorption configurations of Pt_{13} clusters S13

S3 Structural model of $Pt_{cluster}/CeO_2(100)$ S14

S4 O_v location effects in $Pt_7/CeO_2(100)$ systems S15

S5 O_v location effects in $Pt_{13}/CeO_2(100)$ systems S16

S6 Scheme of different polaron position distribution S17

S7 Effect of O_v concentration on adsorption energies of Pt_7/CeO_{2-x} S18

S8 Effect of O_v concentration on adsorption energies of Pt_{13}/CeO_{2-x} S20

S9 Comparison of optimized geometries S21

S10 Adsorption energy on $CeO_2(111)$ surface S21

S11 Pearson correlation of stage 1 . . . . S22

S12 Pearson correlation of stage 2 . . . . S23

S13 GB hyperparameter optimization S24

S14 RF hyperparameter optimization S25

S15 XGBoost hyperparameter optimization S26

S16 ML model performance and feature importance analysis S27

S17 Pt_{19} validation S28

S18 Polaron distribution in Pt_7/CeO_{2-x} S29

S19 Layer-resolved atomic and polaron distribution on Pt_7_flat/CeO_{2-x} models . S30

S20 Polaron distribution in Pt_{13}/CeO_{2-x} S31

S21 Layer-resolved atomic and polaron distribution on Pt_{13}_flat/CeO_{2-x} models . S32

S22 Normalized correlation strengths S32

S23 Charge distribution in Pt_7/CeO_{2-x} S33

S24 Charge variation in Pt_7/CeO_{2-x} S33

S25 Charge distribution in Pt_{13}/CeO_{2-x} S34

S26 Charge variation in Pt_{13}/CeO_{2-x} S35

S27 PDOS analysis of Pt_7/CeO_{2-x} S36

S28 PDOS analysis of Pt_{13}/CeO_{2-x} (3d, bj, and 2l) S37

S29 PDOS analysis of Pt_{13}/CeO_{2-x} (rod and flat) S38

S30 Local atomic RMSD of Pt_7/CeO_{2-x} S39

S31 Local atomic RMSD of Pt_{13}/CeO_{2-x} S40

S32 CO adsorption strength S41

List of Tables

S1 O_v formation energies of $CeO_{2-x}(100)$ S19

S2 Primary feature pool S42

S3 Main characteristics of ML models S43

Supporting Notes

Supporting Note 1

We investigated two representative sizes of platinum clusters, Pt_7 and Pt_{13}, selected to capture the dimensional crossover from planar (2D) to compact (3D) geometries and their distinct structural dynamics reported in previous studies. For Pt_7, we included a planar hexagonal structure and a tetragonal pyramid.[1] For Pt_{13}, we built a planar, a two-layer (2l) structure (inspired by Ni_{13} clusters),[2] a hollow (bj) geometry reported as a global minimum,[3] and a tricapped pentagonal prism structure identified by global optimization.[4]

These structures were placed on $CeO_2(100)$ surfaces at multiple lateral positions and orientations to sample distinct adsorption modes systematically. Local geometry optimization was performed for all configurations. In several cases, surface-induced reconstructions occurred, giving rise to new conformations such as quasi-planar for Pt_7 and rod for Pt_{13}. Therefore, the resulting surface-bound configurations include 2l, quasi-planar (quasi), and flat shapes for Pt_7, and 3d, hollow (bj), 2l, rod, and flat structures for Pt_{13}, which shows in Figures S1 and S2. The variation in adsorption energy (ΔE_{ads}) within each oxygen vacancy (O_v) concentration range also reflects the relative stability of different polaron arrangements. Although an absolute polaron formation energy is not explicitly defined here, the comparison among various polaron distributions provides a consistent measure of their relative stability within the reduced Pt/CeO_{2-x} systems. These structures serve as the basis for all subsequent analyses.

To elucidate the effect of O_v on Pt adsorption behaviour, vacancies were systematically introduced on the $CeO_2(100)$ surface at varying concentrations ranging from 0 to 12.5%. Specifically, we considered seven distinct vacancy concentrations: 0% (pristine surface), 0.78%, 1.56% (only for Pt_{13}), 2.34% (only for Pt_7), 3.91%, 5.47%, 7.03%, and 12.5% (see Figure 1c,d and Figures S7 and S8). Vacancies were introduced by the selective removal of neutral oxygen atoms, followed by structural relaxation. At each vacancy concentration,

comprehensive polaron sampling was performed to systematically explore polaron localization and ensure robust statistical representation of preferred sites. To further verify the robustness of the fluorite lattice, we performed full-cell relaxation test on a random model containing ten polaron sites (3O_v Pt_7_2l/CeO_2(100)). The optimized structure remained fluorite-like without reconstruction, while the total energy decreased by 0.06 eV/atom relative to the fixed-cell relaxation. The lattice parameters changed by +1.88% in x axis and –1.34% in y axis (Figure S9), indicating elastic relaxation rather than any bulk transformation. In addition, a slight modification of the cluster shape from 2l to quasi-planer configuration was observed, further confirming the stability of the extended morphology.

The ΔE_{ads} of the Pt clusters on the ceria support were calculated using the following expression:

$$\Delta E_{\text{ads}} = E_{\text{Pt/CeO}_{2\text{-}x}} - E_{\text{CeO}_{2\text{-}x}} - E_{\text{Pt}_\text{n}^{(\text{gas, stable})}} \tag{S1}$$

where $E_{\text{Pt/CeO}_{2-x}}$ is the total energy of the Pt cluster adsorbed on the ceria surface, $E_{\text{CeO}_{2-x}}$ is the energy of the corresponding pristine or defective ceria surface, and $E_{\text{Pt}_\text{n}^{(\text{gas, stable})}}$ is the energy of the isolated Pt cluster optimized in the gas phase. For the pristine CeO_2(100) surface, the reference state used in Eq. S1 does not contain any polarons. For each cluster size, $E_{\text{Pt}_\text{n}^{(\text{gas, stable})}}$ refers to the total energy of the most stable gas-phase structure of that size, optimized separately. This ensures that all adsorption energies are referenced to a single consistent cluster per size. For each O_v concentration, a single optimized CeO_{2-x} surface is used as the reference. Therefore, $E_{\text{CeO}_{2-x}}$ remains fixed within each O_v level, and the variation in ΔE_{ads} reflects only differences in the stability of the adsorbed Pt cluster configurations. Alternative thermodynamic-cycle schemes (using bulk Pt and pristine CeO_2(100)) are not employed here, as our goal is to isolate the influence of reduction and polaronic effects on the strength of metal–support interactions.

It's worth noting that in this work, oxygen vacancies primarily originate from bulk ceria, surface oxygen vacancies can also form and migrate during catalytic processes. However, our

analysis demonstrates that these vacancies are unlikely to occupy interfacial Pt–O–Ce sites (Figures S21 and S20,[5] thus minimizing their direct destabilizing effect on Pt clusters. This selective vacancy formation mechanism further reinforces the interfacial stability observed in our models.

To assess the facet dependence of the Pt/CeO_2 interaction, we additionally examined Pt_7 and Pt_{13} clusters supported on CeO_2(111) (Figure S10). Both cluster sizes show relatively minor variations in ΔE_{ads} as the O_v concentration increases, in contrast to the pronounced decrease observed on CeO_2(100). However, the flat-shaped Pt_{13} cluster still shows the largest change in ΔE_{ads} ($\Delta\Delta E_{\text{ads}} = 2.0$ eV), indicating that this morphology is the most responsive to substrate reduction. This overall weak dependence can be attributed to the much higher O_v formation energy (2.77 eV) and the limited mobility of Ce^{3+} polarons on the (111) surface, which together suppress electron exchange between the oxide and the metal cluster. Consequently, the Pt/CeO_2(111) interface remains structurally and electronically inert even under reducing conditions, whereas CeO_2(100) exhibits strong redox-driven coupling, as discussed in the main text.

Supporting Note 2

In order to better understand the effect of O_v position on the stability of the adsorption of Pt clusters, we have systematically studied the surface, subsurface, and bulk O_v sites in Pt_7/CeO_2(100) and Pt_{13}/CeO_2(100). In both cases of cluster sizes, our results (Figures S4 and S5) are less clear when varying the depth of a vacancy. In particular, there was no uniform or significant stabilization due to the vacancies being purely located in any region considered (surface, subsurface, or bulk). This finding implies that, for the conditions studied, the exact depth of the vacancy has little effect on the stability of Pt cluster adsorption. Hence, defect-engineering strategies in the future for tuning the catalysts' properties should be more concerned with structural or electronic factors rather than unsaturated depths alone. We also examined the relative stability of different polaron configurations generated from 1

subsurface O_v on $CeO_2(100)$. Three representative configurations were considered in Figure S6: nearest neighbour (NN), next-nearest neighbour (NNN), and more distant (far) sites relative to the vacancy. The results show that the configuration with two polarons localized at the NN position is the most stable, whereas the NNN and far configurations are higher in energy by 0.07 and 0.13 eV, respectively. The small energy differences indicate that polaron migration is energetically feasible, in line with previous studies.[6,7]

Supporting Note 3

To elucidate the structure–property relationships governing Pt/CeO_{2-x} catalytic systems, we curated a diverse set of physicochemical features encompassing the Pt cluster, Pt–O interface, and CeO_{2-x} support domains. These features were systematically extracted from DFT optimized structures and are detailed in Table S2.

Pt Cluster Domain: The structural distortion of Pt clusters upon adsorption was quantified using the root mean square deviation (RMSD) from their gas-phase optimized geometries. This metric effectively captures the extent of geometric perturbation induced by metal–support interactions. Charge transfer (Δq) between the Pt cluster and the CeO_{2-x} support was evaluated through Bader charge analysis, providing insights into electronic metal–support interactions (EMSIs) that are crucial in modulating catalytic activity.

Pt–O Interface Domain: features in this category encompass statistical measures of Pt–O bond lengths, including minimum, maximum, mean, and standard deviation values, as well as the total count and cumulative length of Pt–O bonds. These features characterize the coordination environment at the metal–oxide interface, which is critical for understanding adsorption phenomena and catalytic performance.

CeO_{2-x} Support Domain: To capture the redox dynamics of the ceria support, we included features such as O_v concentration and polaron-related features. The number and spatial distribution of Ce^{3+} sites (polarons) were determined based on localized magnetic moments, employing a threshold of 0.8 μ_B, consistent with established criteria.[8,9]

Polaron-related descriptors include:

- *Polaron–polaron interaction energy* ($E_{\text{pol-pol}}$), representing the Coulombic repulsion between Ce^{3+} sites:

$$E_{\text{pol-pol}} = \sum_{i<j} \frac{1}{\varepsilon_r \cdot r_{ij}} \tag{S2}$$

  where r_{ij} is the distance between Ce^{3+} ions i and j, and ε_r is an effective dielectric constant for reduced ceria, intermediate between the bulk value and local screening effects near oxygen vacancies.[10,11]

- *Polaron–lattice interaction energy* ($E_{\text{pol-lattice}}$), describing the elastic energy associated with polaron displacement:

$$E_{\text{pol-lattice}} = \sum_{i=1}^{N} k \cdot |\mathbf{r}_i - \mathbf{r}_0|^2 \tag{S3}$$

  where $k = 1.0$ is a harmonic force constant (used for relative comparison), $\mathbf{r}_i$ is the position of the i-th Ce^{3+}, and $\mathbf{r}_0$ is the geometric center of all Ce^{3+} positions.

- *Surface strain* (ε), quantifying the average number of surface oxygen atoms (top 20% by z-coordinate) within 2.5 Å of each Ce^{3+} site, providing a measure of local coordination environment and lattice distortion.

Additional features include Ce^{3+}–Ce^{3+} pairwise distance statistics (sum, minimum, mean, maximum, standard deviation) to account for the influence of defect structures and electronic localization on catalytic behaviour. Besides, feature selection was guided by Pearson correlation coefficients and agglomerative clustering to identify and mitigate multicollinearity, ensuring the robustness of subsequent machine learning models.

Supporting Note 4

The machine learning (ML) workflow was implemented as an open-source Python toolkit specifically developed for analyzing metal-supported oxide catalysts. The framework is mod-

ular and fully reproducible, featuring a centralized entry point (run_analysis) that orchestrates all steps. Core Python libraries used include scikit-learn $\geq$1.3 (model training and optimization),[12] Pandas $\geq$1.3 (data processing),[13] Matplotlib $\geq$3.4 and Seaborn $\geq$0.11 (visualization),[14,15] and SciPy $\geq$1.7 (statistical analysis).[16] Configuration parameters are systematically defined and maintained in a dedicated control file (config.py), ensuring clarity and facilitating reproducibility.

Model hyperparameters were optimized using a two-stage tuning approach. Initially, a comprehensive grid search (GridSearchCV, scikit-learn $\geq$1.3)[12] explored the hyperparameter space broadly to identify parameter regions. Subsequently, a Bayesian optimization strategy (BayesSearchCV from scikit-optimize 0.9.0)[17] refined these parameters through iterative probabilistic sampling, efficiently converging towards optimal values. Figures S13- S15 summarize the variation of model accuracy metrics (R^2, RMSE, and MAE) as a function of these parameters. The results indicate that all three models exhibit stable predictive performance within the optimal parameter ranges, confirming that the ML results discussed in the main text are robust with respect to reasonable parameter variations.

Stage 1 of the workflow employs original features directly extracted from DFT-relaxed Pt/CeO_{2-x} structures in three main domains (Supporting Note 3). Machine-learning models trained on these standardized features included Gradient Boosting (GB),[18] Random Forest (RF),[19] and XGBoost regressors.[20] Model performance was validated using five-fold cross-validation. These three models were chosen because they represent complementary, well-established tree-based ensemble methods that balance interpretability, robustness, and the ability to capture nonlinear structure–property relationships. Their main learning strategies, characteristics, advantages, and limitations are summarized in Table S3. In Stage 2, physically meaningful high-order features were constructed using pairwise operations among the original features. A correlation-based filtering step selected features most strongly associated with the targeted feature, which in this work is ΔE_{ads}.

To assess inter-feature dependencies, we constructed a Pearson correlation network among

selected features using SciPy ≥1.7.[16] Pearson correlation measures the strength of linear relationships between feature pairs, with values ranging from −1 (perfect negative correlation) to +1 (perfect positive correlation). This analysis serves two purposes: (1) identifying redundant features for model simplification, and (2) revealing physically meaningful couplings between properties. Besides, hierarchical agglomerative clustering grouped similar features based on their correlation structure, and representative descriptors were selected from each cluster to reduce redundancy. Furthermore, Bonferroni correction is utilized here to identify statistically significant connections (Bonferroni-adjusted $p < 0.05$). The Bonferroni correction is a multiple testing correction method that adjusts the significance threshold to control the family-wise error rate when performing numerous statistical tests simultaneously. When testing correlations among our feature set, the number of pairwise comparisons increases substantially. For instance, 19 original features generate 171 unique pairwise correlations, raising the risk of identifying spurious relationships by chance. Specifically, the Bonferroni method adjusts the significance threshold to α/n, where α is the desired overall significance level (typically 0.05) and n is the number of independent tests. This approach ensures that only robust, physically meaningful correlations are retained in the network.

Supporting Note 5

For quantitative comparison of PDOS across models that differ in structure or electronic character, we utilized a general alignment procedure with the O 2s band centre as the universal energy reference. This method clearly presents an advantage with respect to Fermi-level based alignment ($E - E_{\mathrm{F}}$), where the alignment can be sensitive to system-dependent changes such as defect concentration, surface termination, or slab thickness.[21]

Computational protocol:

- For each structure, the atomic positions were read from the structure file and the central layer oxygen atoms identified by their z-coordinates.

- The PDOS for O 2s orbitals of these reference oxygen atoms was calculated, and the O 2s band centre was determined by integrating the PDOS in the energy window of the O 2s peak.

- All PDOS data for each system (including Fermi level) were shifted so that their O 2s band centre matches that of the pristine CeO_2 reference (defined as $E = 0$ eV).

- The resulting aligned PDOS curves allow direct comparison of band edges, defect/adsorbate states, and Fermi levels across all models, independent of system-specific shifts in absolute energy.

This protocol was scripted in-house in Python, and all PDOS analyses in this work were performed by this protocol. The results are shown in Figures S27– S29.

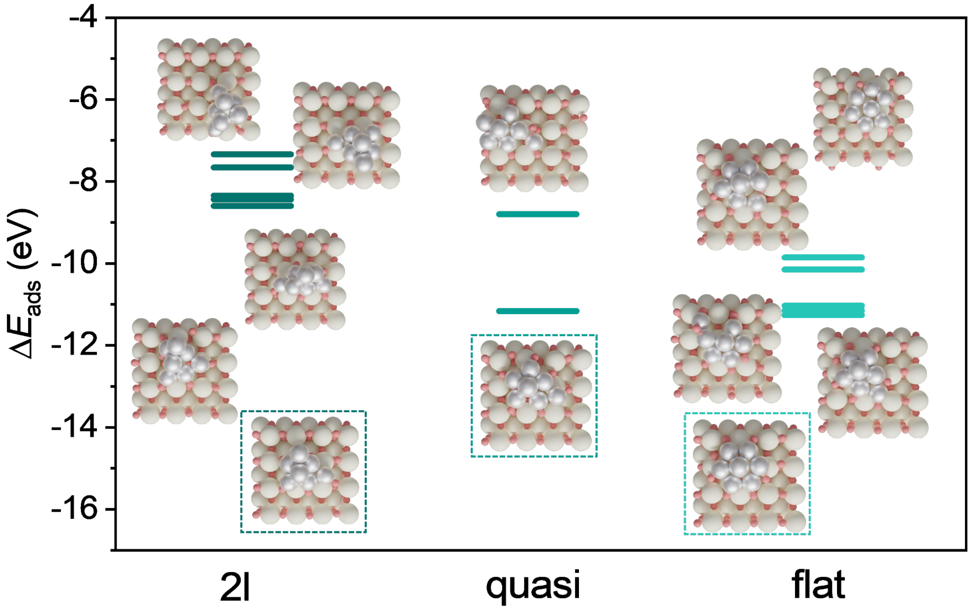

Figure S1: Adsorption energy and structural models of Pt_7 cluster configurations on the (100) surface of ceria. The configurations are ordered from top to bottom according to decreasing adsorption energy. Models outlined with dashed lines indicate the structures selected for further investigation. Colour code: Ce, white; O, pink; Pt cluster, silver.

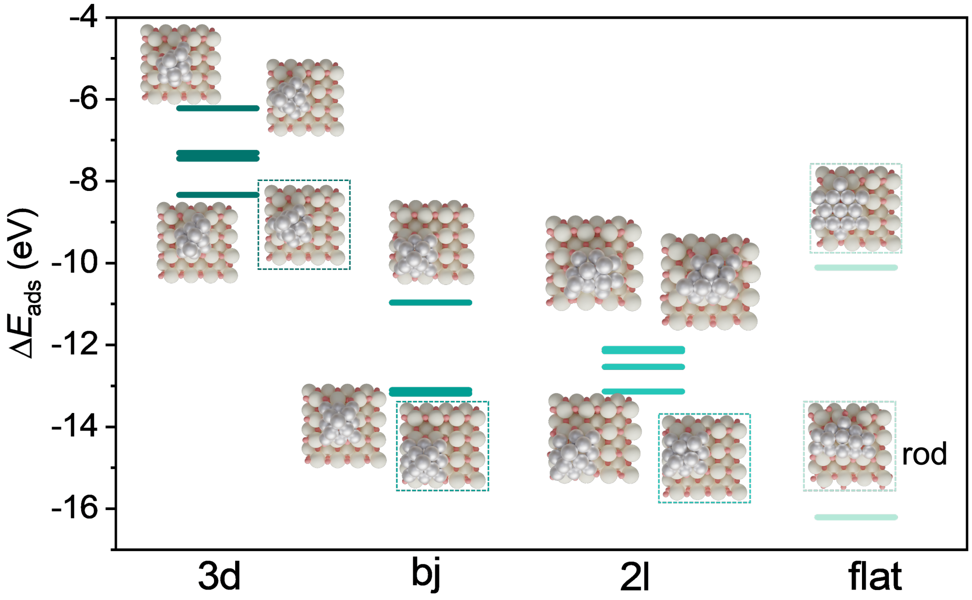

Figure S2: Adsorption energy and structural models of Pt_{13} cluster configurations on the (100) surface of ceria. The configurations are ordered from top to bottom according to decreasing adsorption energy. Models outlined with dashed lines indicate the structures selected for further investigation.

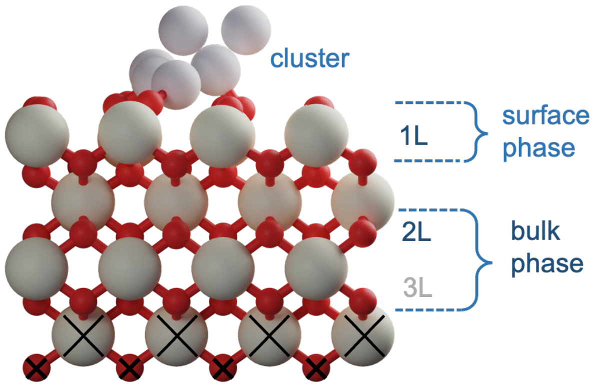

Figure S3: Structural configurations of Pt clusters on $CeO_2(100)$. The ceria support is divided into surface and bulk phases, with the bulk region being the primary site of oxygen vacancy (O_v) formation. In this work, two strategies were used to generate initial oxygen vacancies: the first considers vacancies formed only within the second layer (2L), while the second includes vacancies in both the second and third layers (2L and 3L). It is important to note that after polaron sampling, the final positions of the oxygen vacancies may differ from their initial configurations. A fixed bottom layer is included to maintain structural stability, but is not considered in the defect engineering analysis of this study.

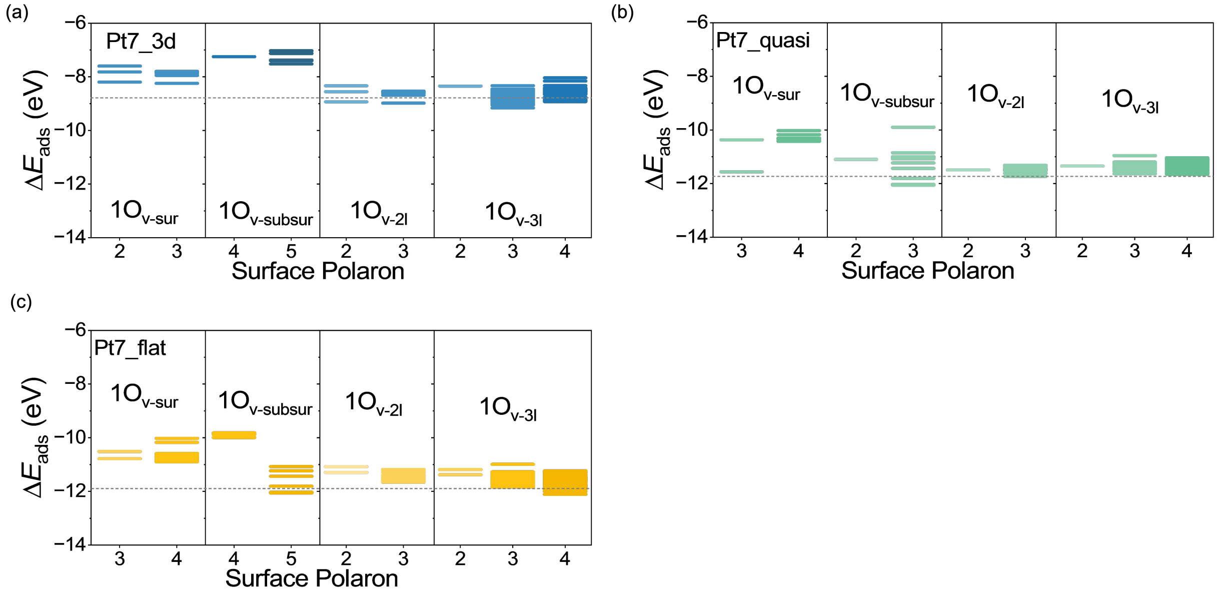

Figure S4: O_v location effects in $Pt_7/CeO_2(100)$ systems. The results reveal only minor variations between vacancies located at surface, subsurface, and bulk positions. There was no clear or significant stabilization effect associated exclusively with vacancies in a specific location. These findings suggest that, under the investigated conditions, the adsorption stability of Pt clusters exhibits limited dependence on the precise location of oxygen vacancies.

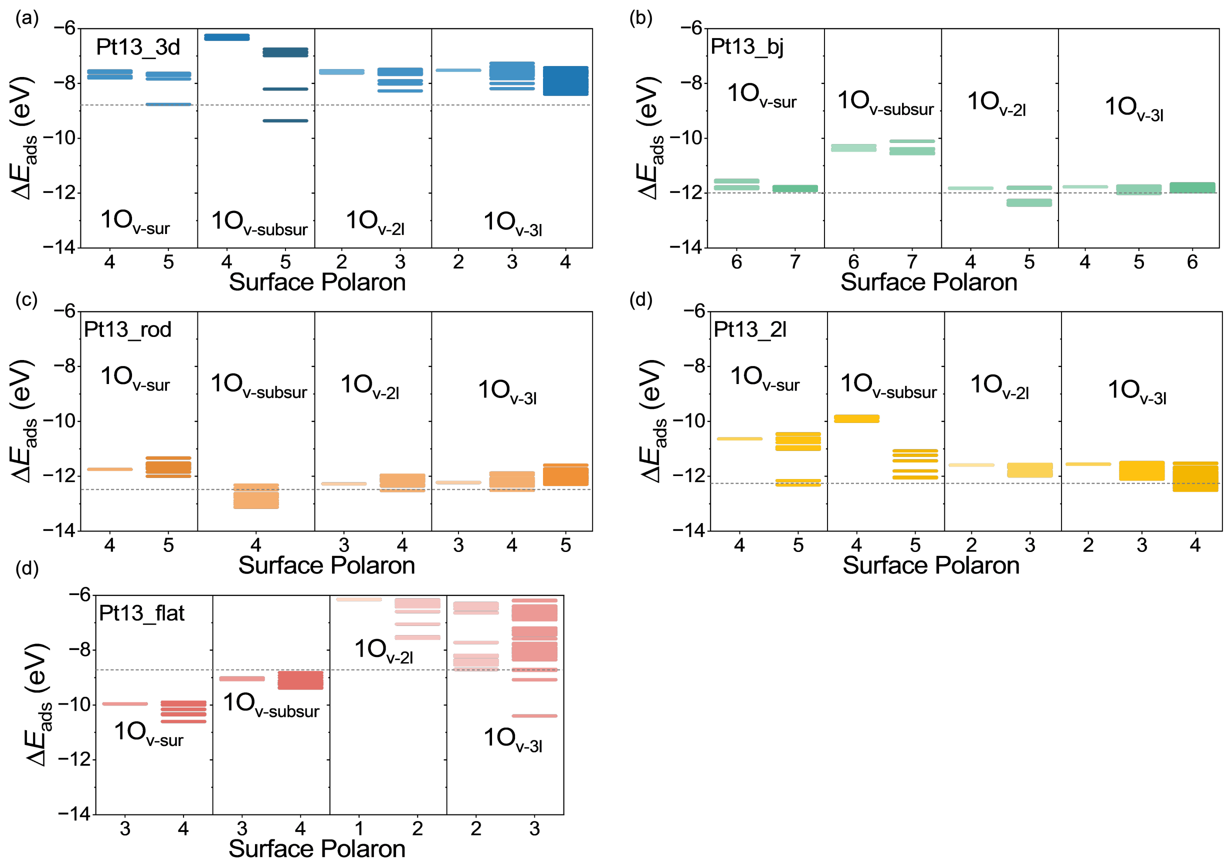

Figure S5: O_v location effects in $Pt_{13}/CeO_2(100)$ systems. The adsorption energies for Pt_{13} clusters exhibit considerable variability and overlap across oxygen vacancies located at the surface, subsurface, and deeper bulk regions. The absence of clear trends or systematic differences indicates that Pt_{13} cluster adsorption is not strongly or consistently influenced by the location of oxygen vacancies under these conditions. This suggests a higher degree of complexity or randomness in the adsorption behaviour of larger clusters, implying a relatively weak dependence or sensitivity of Pt_{13} adsorption stability on oxygen vacancy positions.

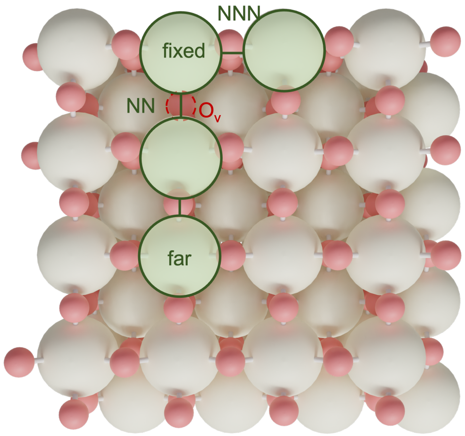

Figure S6: Schematic illustration of different polaron configurations relative to 1 subsurface O_v in CeO_2. One Ce^{3+} site is fixed, while the second polaron is positioned at NN, NNN, and far Ce sites.

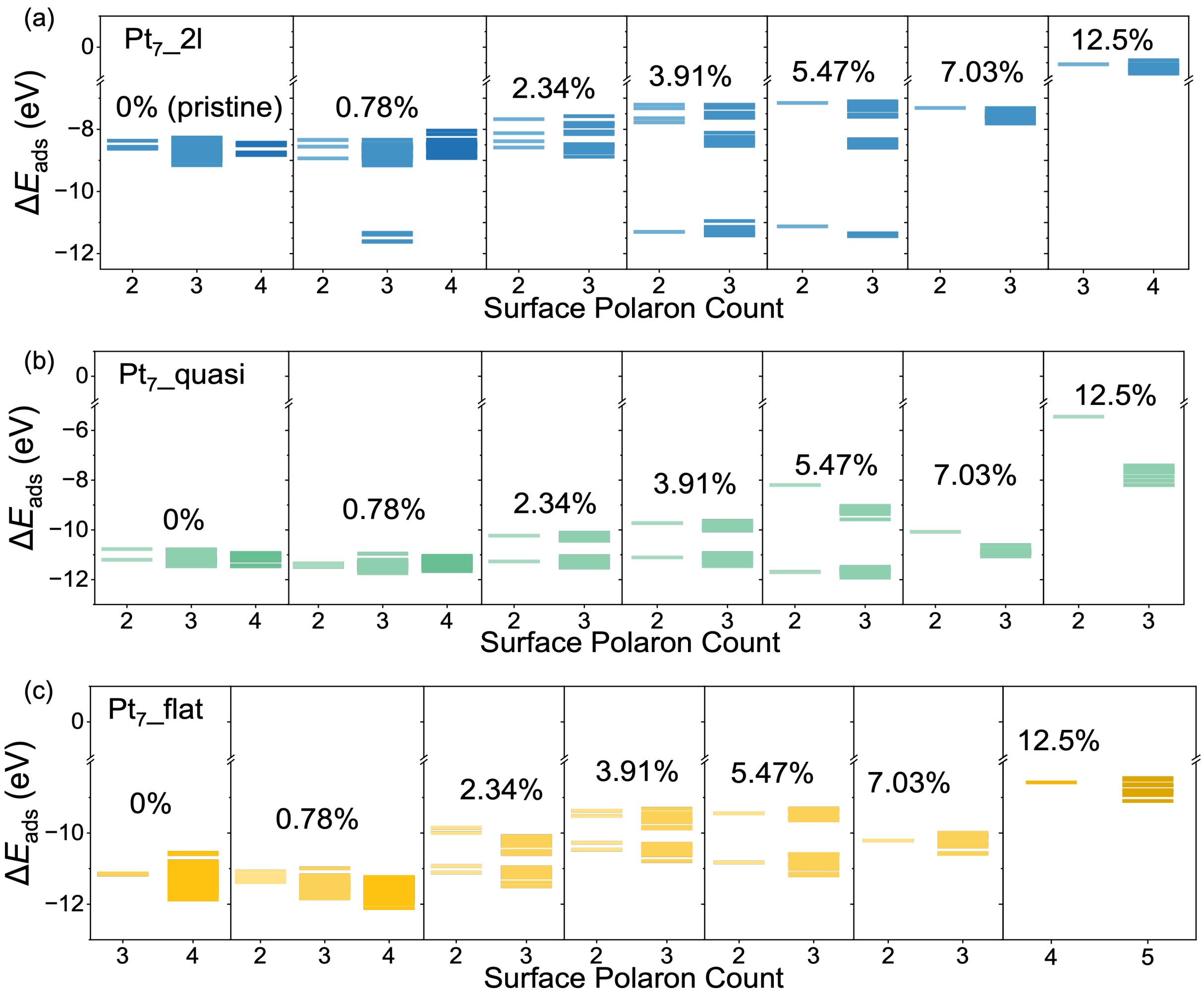

Figure S7: Effect of O_v concentration and surface polaron count on adsorption energies of Pt_7/CeO_{2-x}.

Table S1: O_v formation energies (referenced by H_2O and H_2) of $CeO_{2-x}(100)$ at various defect concentrations. $\Delta E_{\mathrm{diff(Ov)}}$ denotes the differential formation energy of the corresponding O_v, and ΔE_{Ov} the overall formation energy.

**O_v concentration/ %**	$\Delta E_{\mathrm{diff}}(\mathrm{Ov})$ **/ eV**	ΔE_{Ov} **/ eV**
0.78	−0.46	−0.46
2.34	−1.42	−0.63
3.91	−0.62	−0.50
5.47	−1.39	−0.56
7.03	+2.19	−0.19
12.50	−0.94	−0.16

Table S1 shows that even at relatively high vacancy concentrations (up to 12.5%), the energies remain within thermodynamically feasible ranges, indicating that O_v formation on $CeO_2(100)$ is energetically possible by varying the external reservoir oxygen pressure.

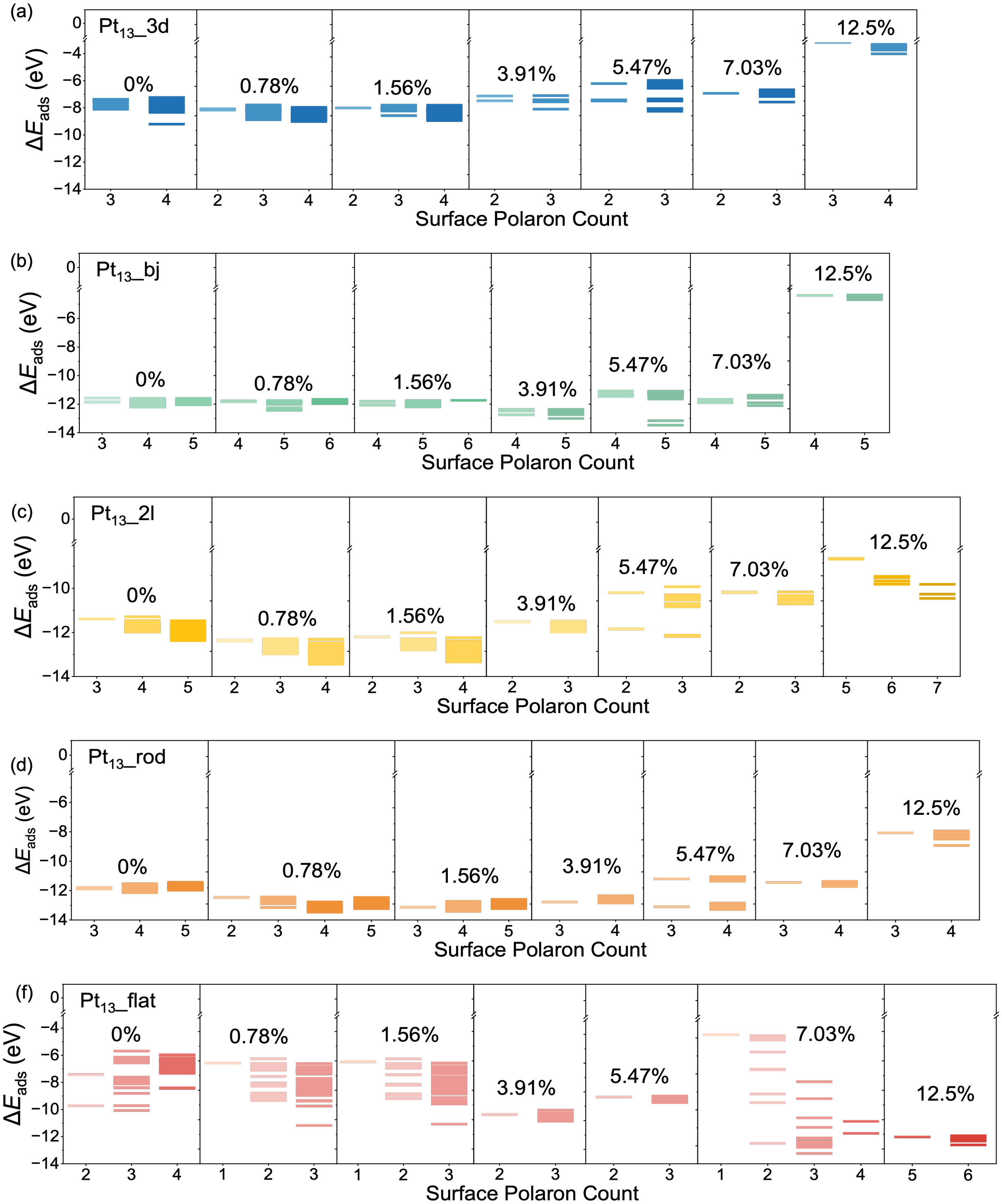

Figure S8: Effect of O_v concentration and surface polaron count on adsorption energies of Pt_{13}/CeO_{2-x}.

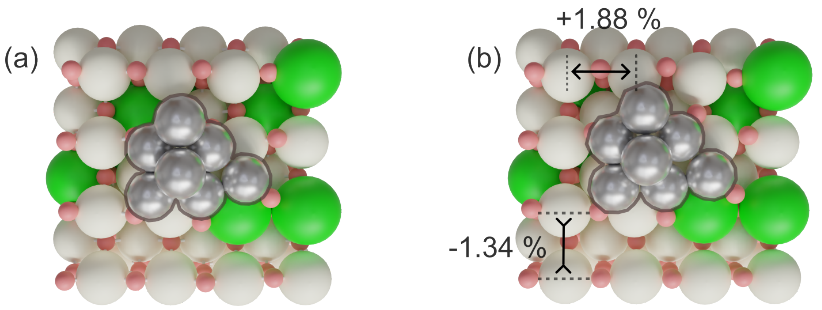

Figure S9: Comparison of the optimized geometries of Pt_7_3d/CeO_{2-x} with ten polaron sites: (a) optimized with fixed cell size, and (b) optimized without cell size constraint.

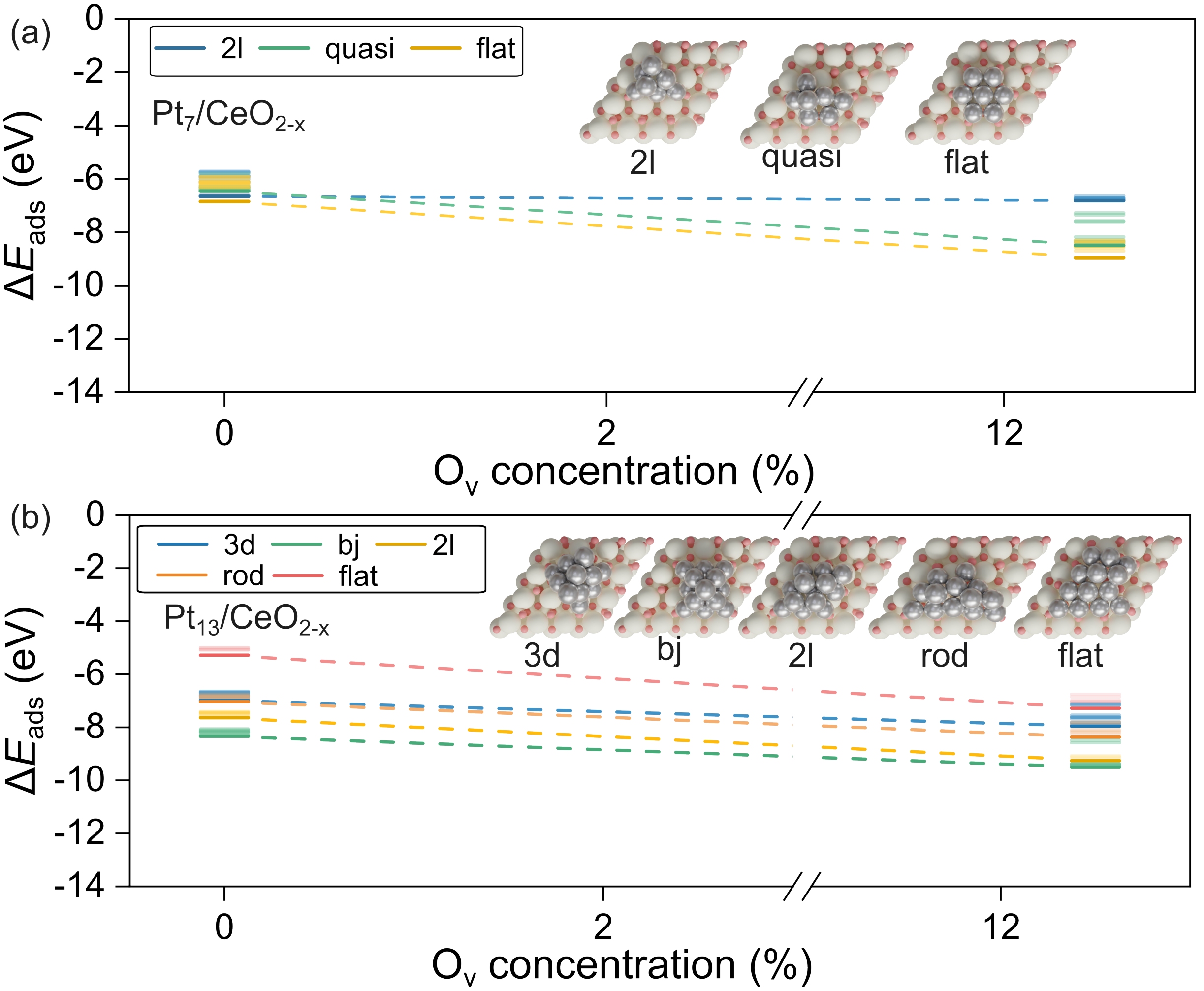

Figure S10: (a) ΔE_{ads} of Pt_7/CeO_{2-x} and (b) Pt_{13}/CeO_{2-x} as a function of O_v concentration on the (111) surface.

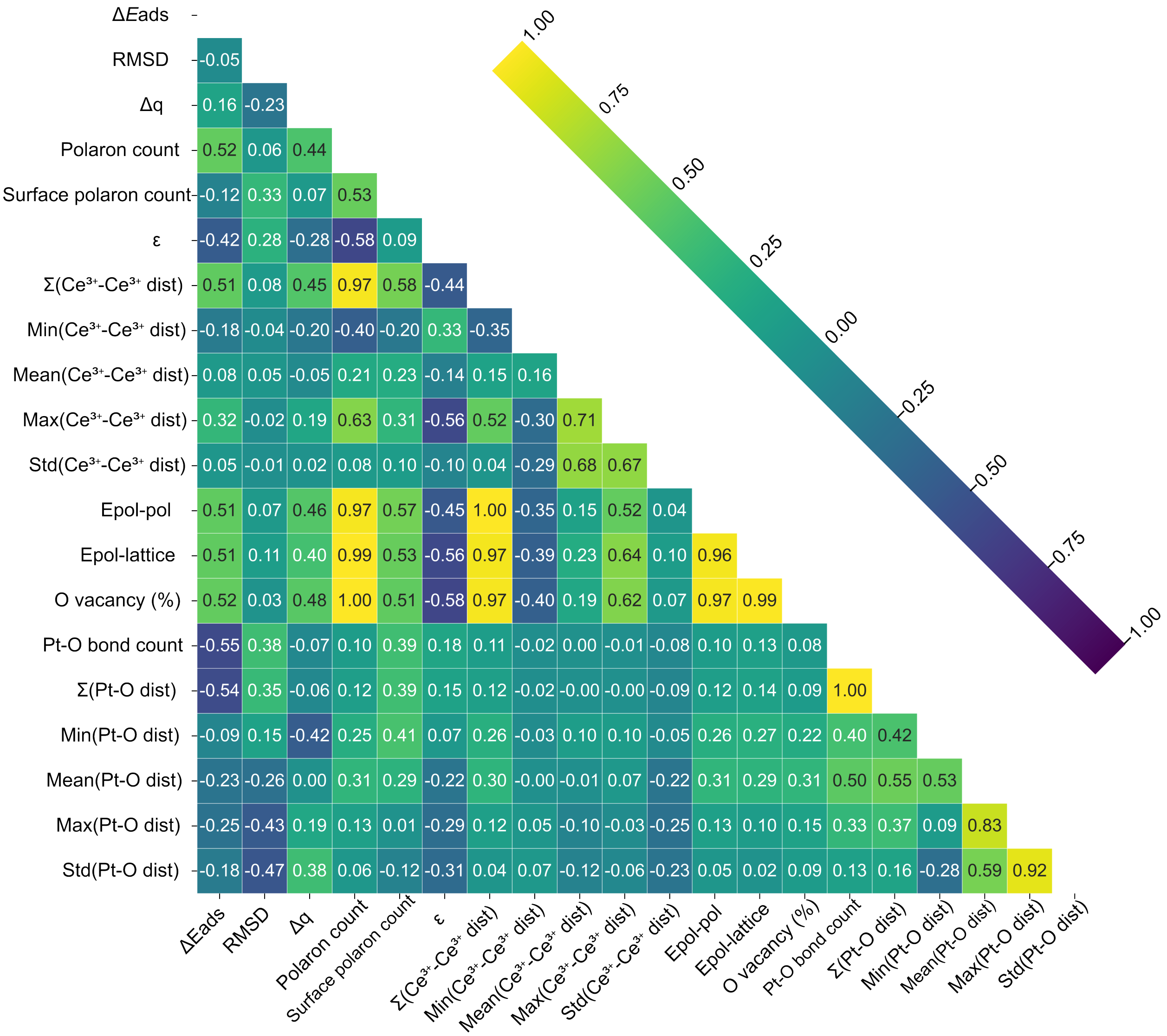

Figure S11: Stage 1 Pearson correlation matrix for all original features. Primary features of the pool are listed in Table S2.

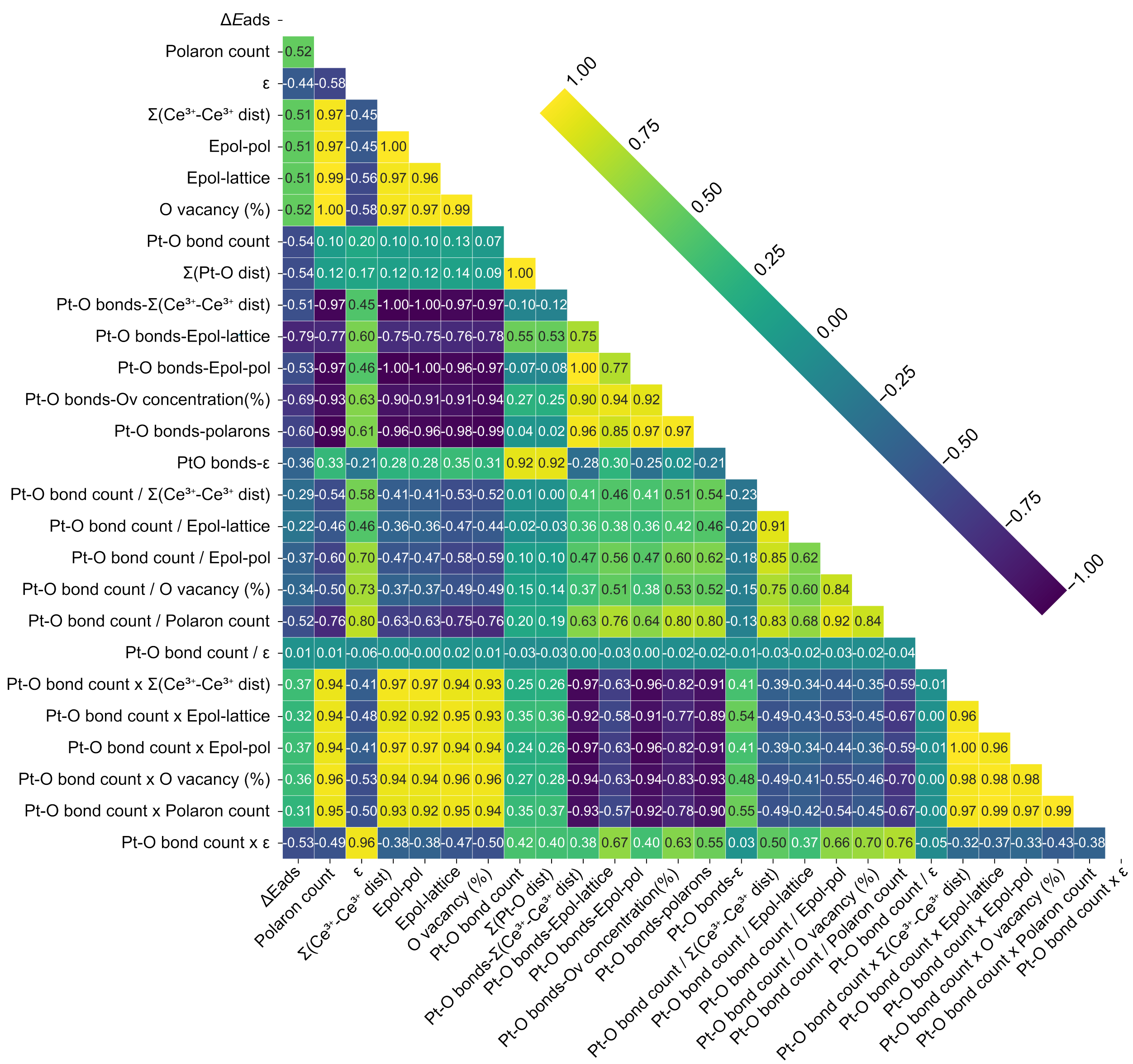

Figure S12: Stage 2 Pearson correlation matrix for all original features.

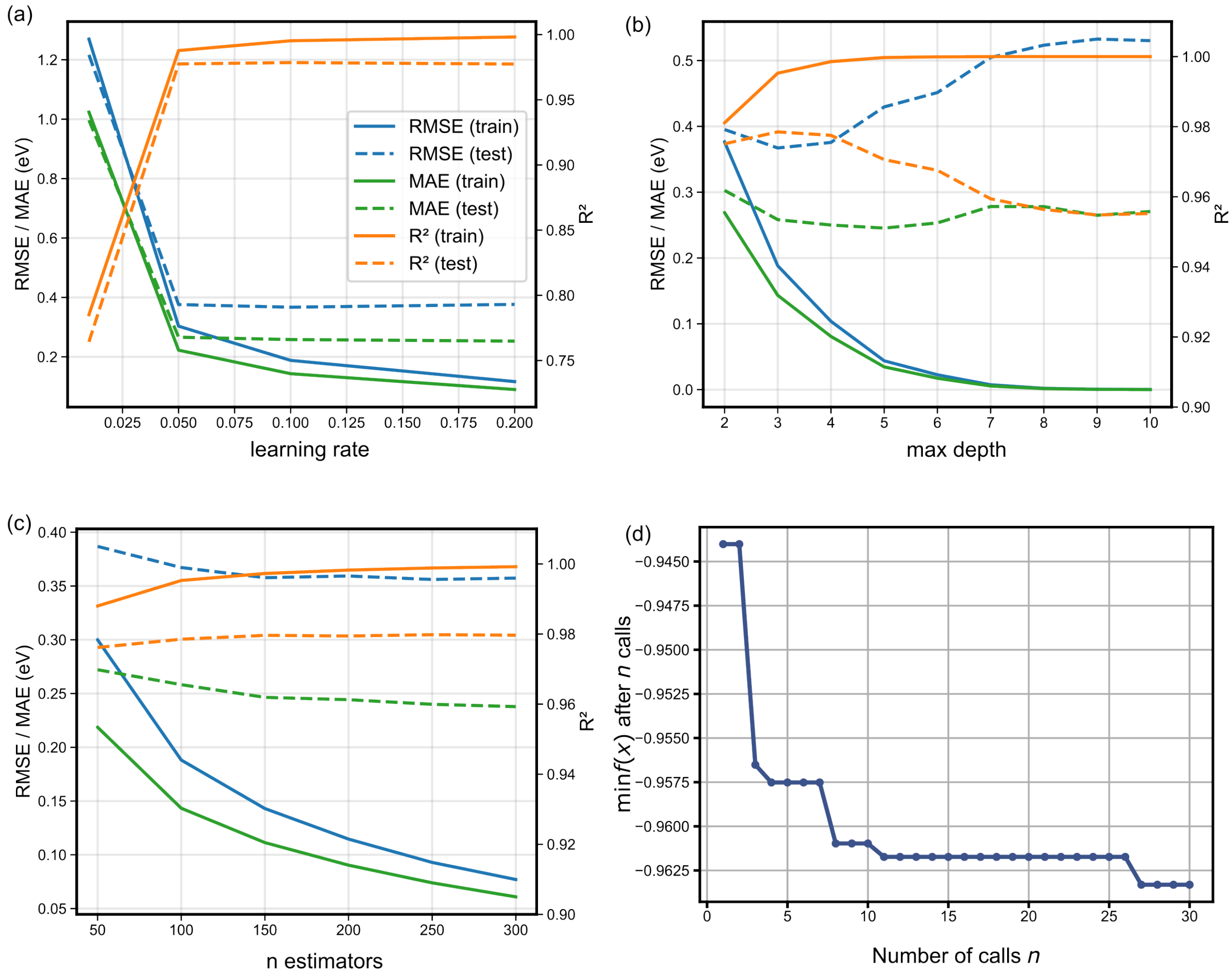

Figure S13: GB hyperparameter optimization. (a–c) Dependence of model performance on learning rate, maximum tree depth, and number of estimators, respectively. R^2 (right axis) and RMSE/MAE (left axis) are shown for both training and test sets under five-fold cross-validation. (d) Convergence trace of the Bayesian optimization, showing the evolution of the best-achieved objective (R^2) with the number of optimization calls.

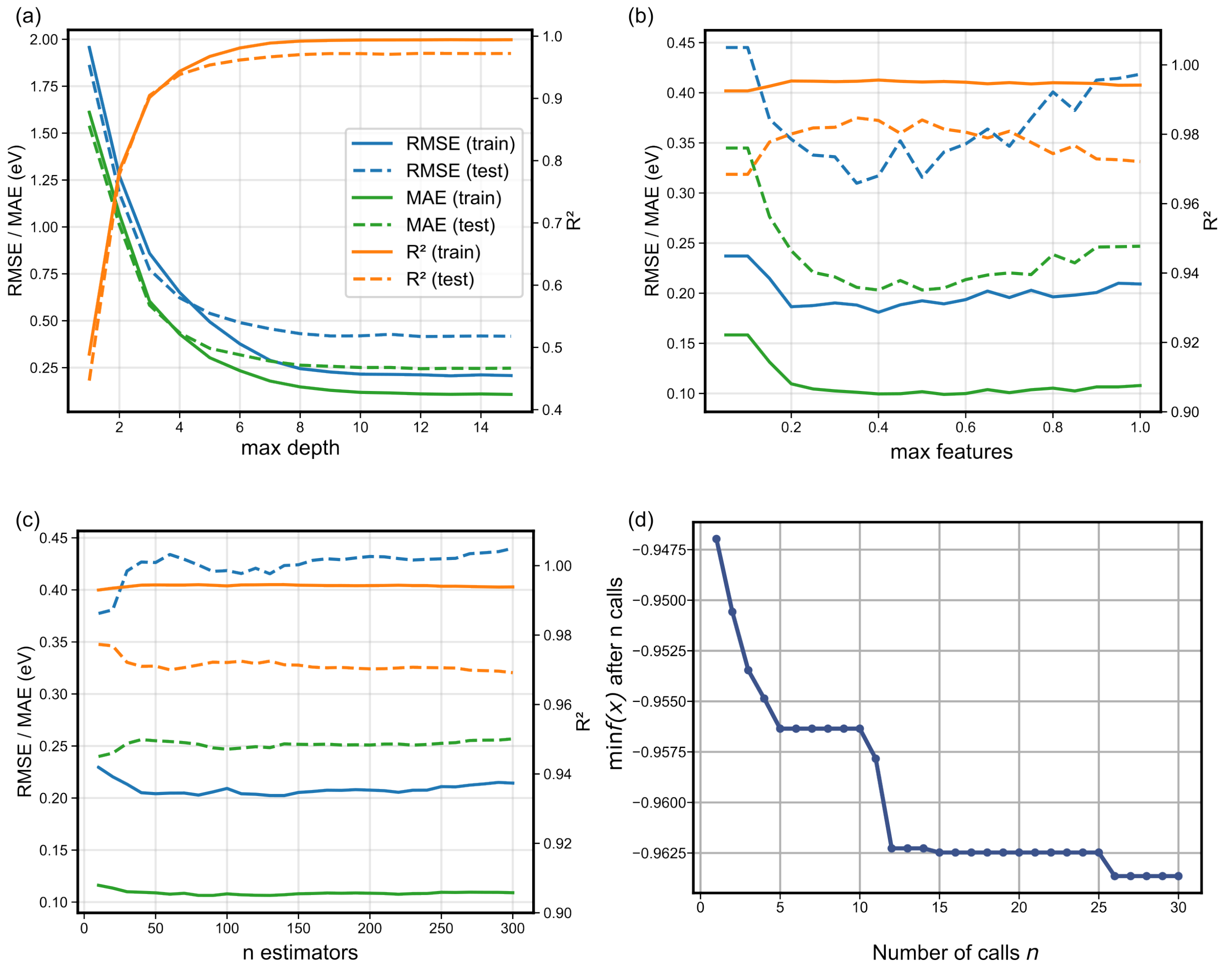

Figure S14: RF hyperparameter optimization. (a-c) Dependence of model performance on maximum tree depth, maximum feature fraction, and number of estimators, respectively. (d) Convergence trace of the Bayesian optimization, showing the evolution of the best-achieved objective (R^2) with the number of optimization calls.

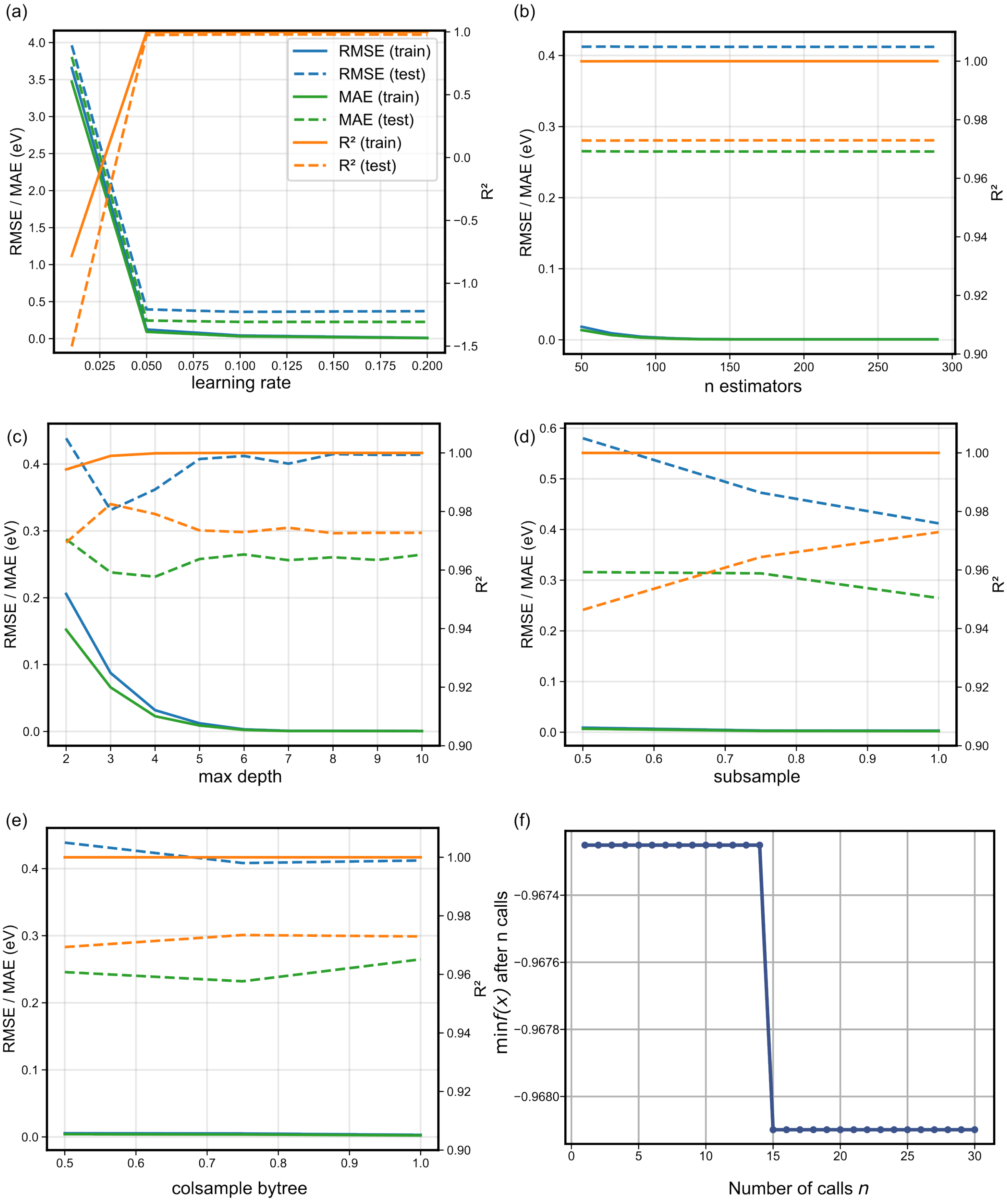

Figure S15: XGBoost hyperparameter optimization. (a-e) Dependence of model performance on (a) learning rate, (b) number of boosting rounds (n estimators), (c) maximum tree depth, (d) subsample ratio of training instances (subsample), and (e) feature subsample ratio per tree (colsample bytree). (f) Convergence trace of Bayesian optimization, showing the evolution of the best-achieved objective (R^2) with the number of optimization calls.

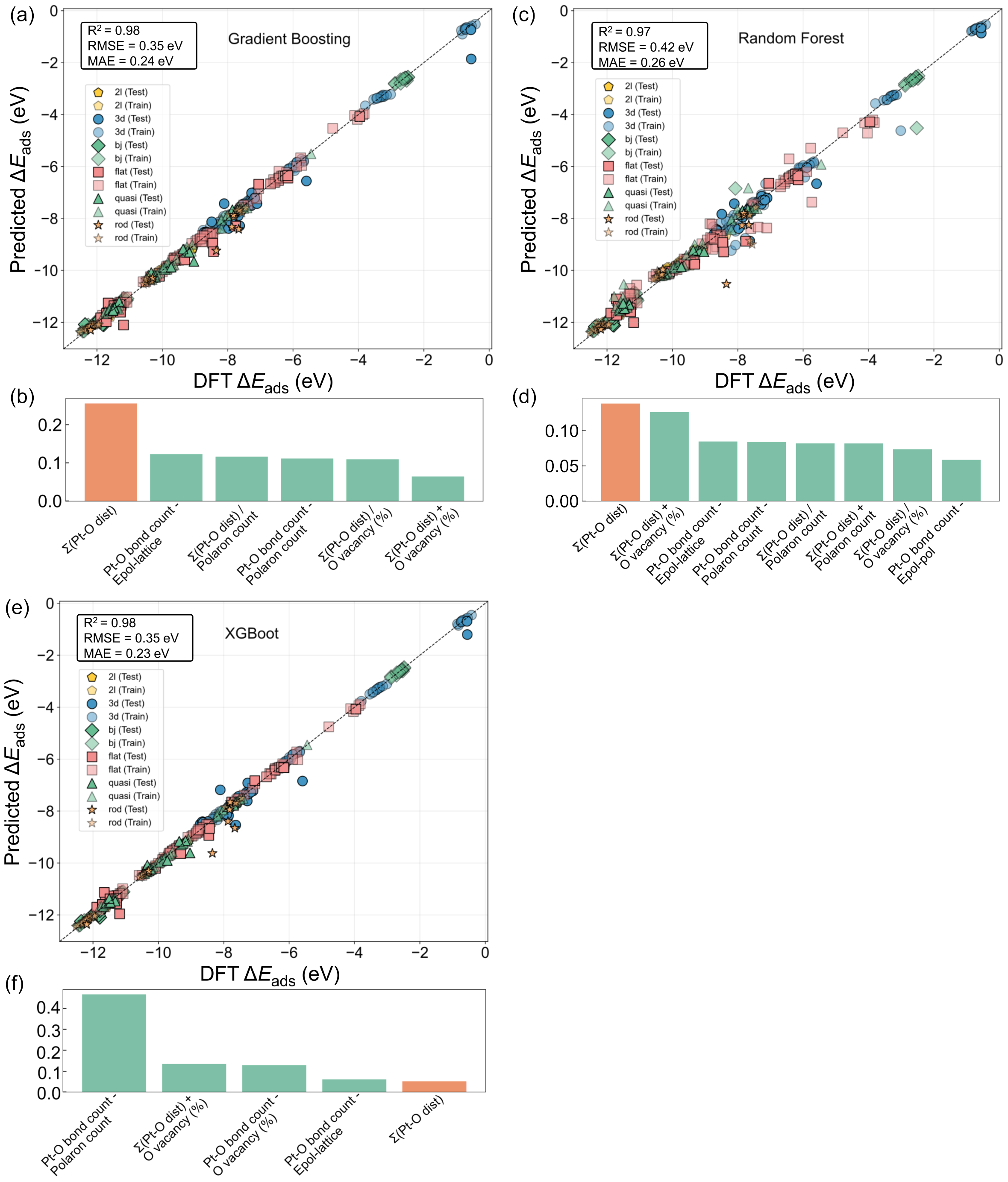

Figure S16: Parity plots (top row) show the predicted versus DFT-computed adsorption energies (ΔE_{ads}) for the test and training sets using (a) GB, (b) RF, and (c) XGBoost regression models trained on physically constructed features. Each point is coloured and shaped by cluster geometry and data split (train/test). R^2, RMSE, and MAE values for each model are annotated in the legend. Bar plots (bottom row) display the relative importance of the top-ranked features selected by each model in Stage 2.

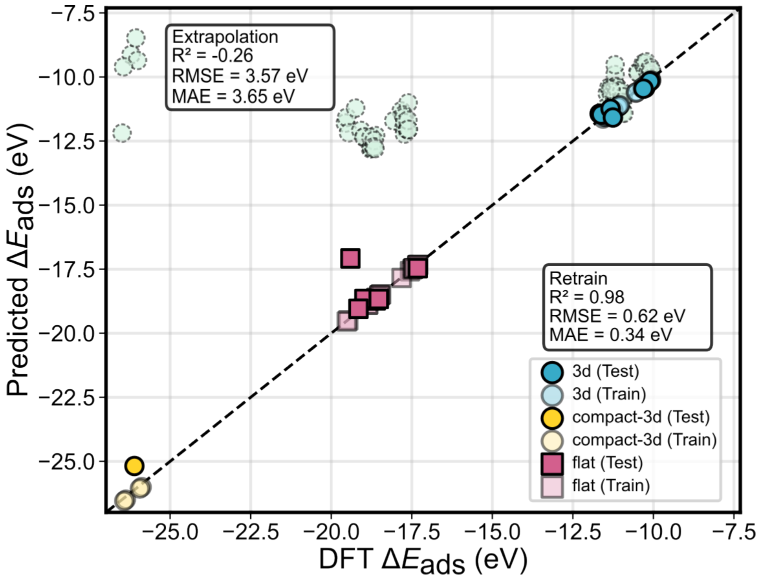

Figure S17: Cross-size validation and retrain performance of the GB model for $Pt_{19}/CeO_2(100)$. (Light) blue circles correspond to 3d structures, (light) yellow circles correspond to compact-3d structures, and (light) pink squares correspond to flat structures (train/test). Light green points represent the extrapolated predictions from the Pt_7/Pt_{13} model.

When extended to the larger 5×5 $CeO_2(100)$ supercell and varying oxygen-vacancy concentrations (pristine and 1% bulk vacancies) with Pt_{19} clusters, direct extrapolation from the Pt_7/Pt_{13} model yields limited accuracy yielded limited predictive accuracy ($R^2 \approx -0.26$) due to configurations lying outside the original energy domain. Retraining with 62 $Pt_{19}/CeO_2(100)$ configurations (80:20 train/test split) rapidly restores predictive performance with ($R^2 > 0.98$), highlighting the importance of efficient dataset preparation and fine-tuning in ML workflows.

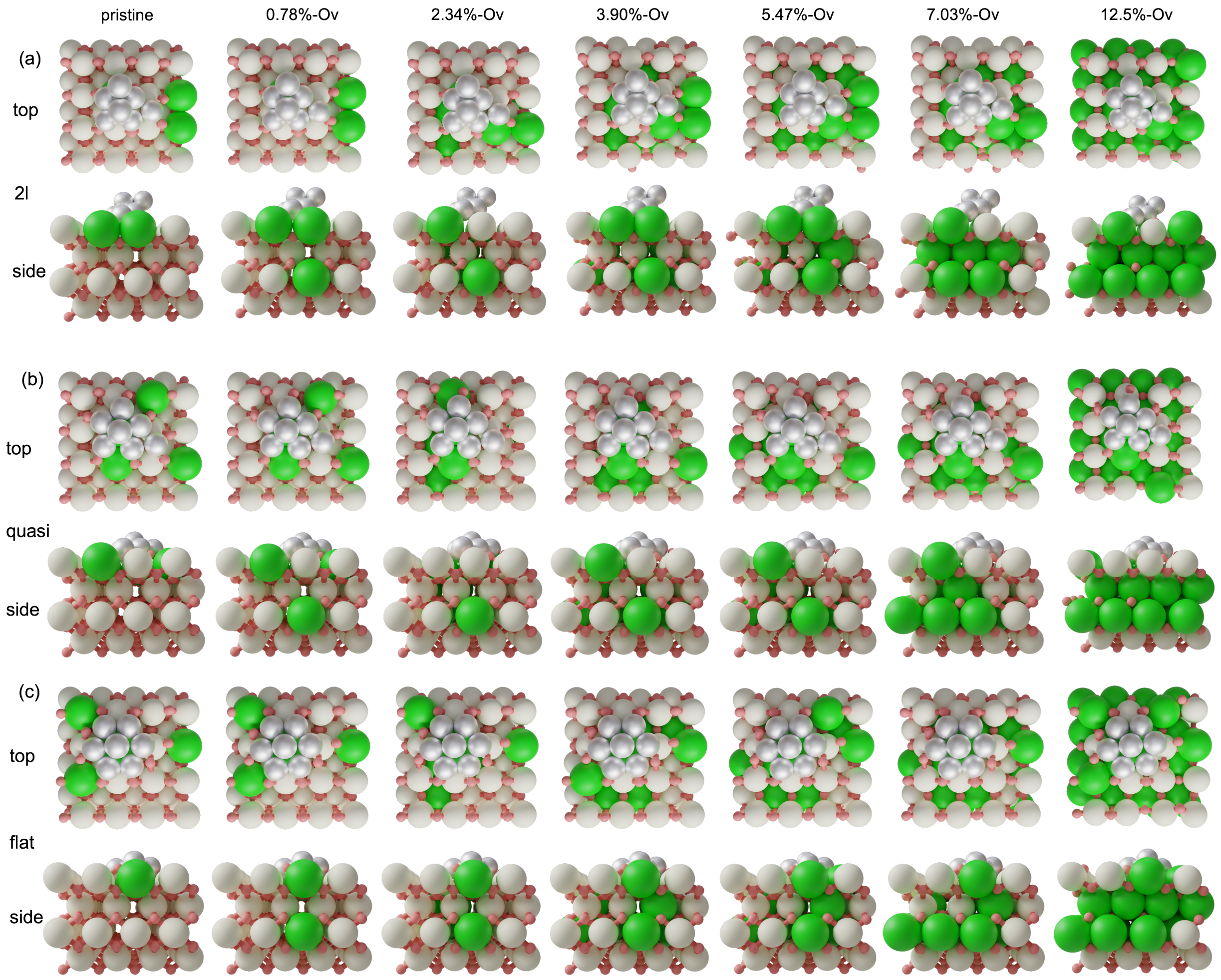

Figure S18: Representative optimized structures of Pt_7/CeO_{2-x} models with diverse O_v and polaron configurations. (a) Pt_7_2l/CeO_{2-x}. (b) Pt_7_quasi/CeO_{2-x}. (c) Pt_7_flat/CeO_{2-x}. Each panel shows a unique sampling of O_v and Ce^{3+} (polaron) distributions. Top and side views are shown for each configuration. Pt clusters are coloured silver, Ce^{4+} atoms are white, O atoms are pink, and Ce^{3+} sites are highlighted in green.

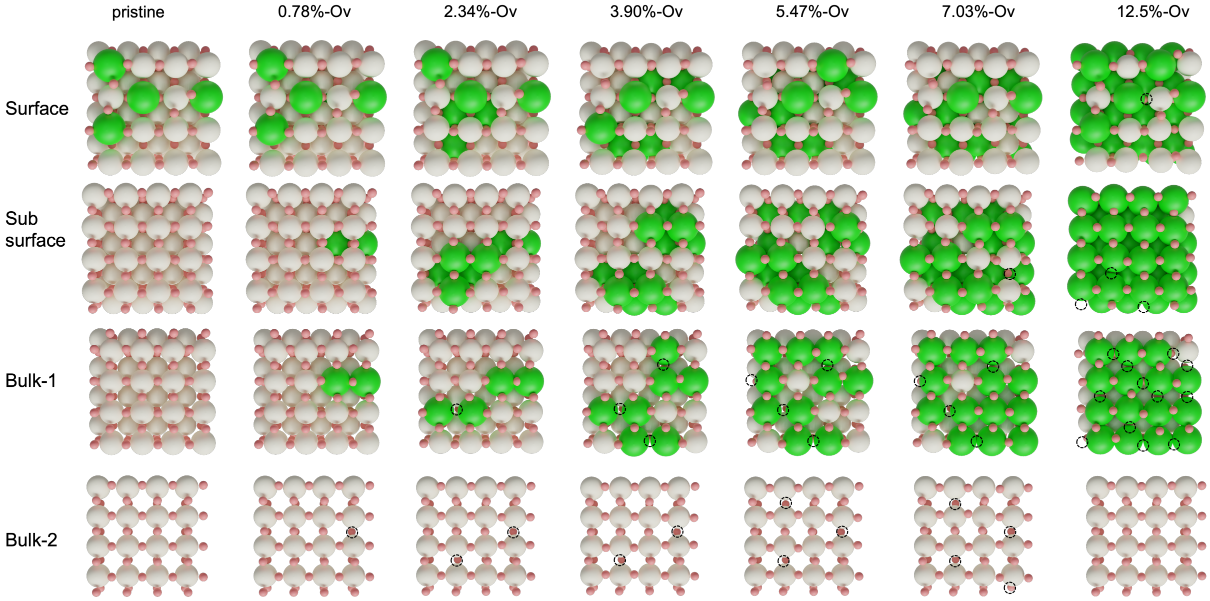

Figure S19: Layer-resolved visualization of oxygen vacancy positions and polaron distributions for the Pt_7_flat/CeO_{2-x} model at different oxygen vacancy concentrations. From top to bottom, the surface, subsurface, first bulk, and second bulk layers are shown separately. Oxygen vacancies are indicated by dashed circles, and Ce^{3+} sites associated with localized polarons are highlighted in green. This representation allows direct identification of the spatial relationship between oxygen vacancies and polaron localization across different layers.

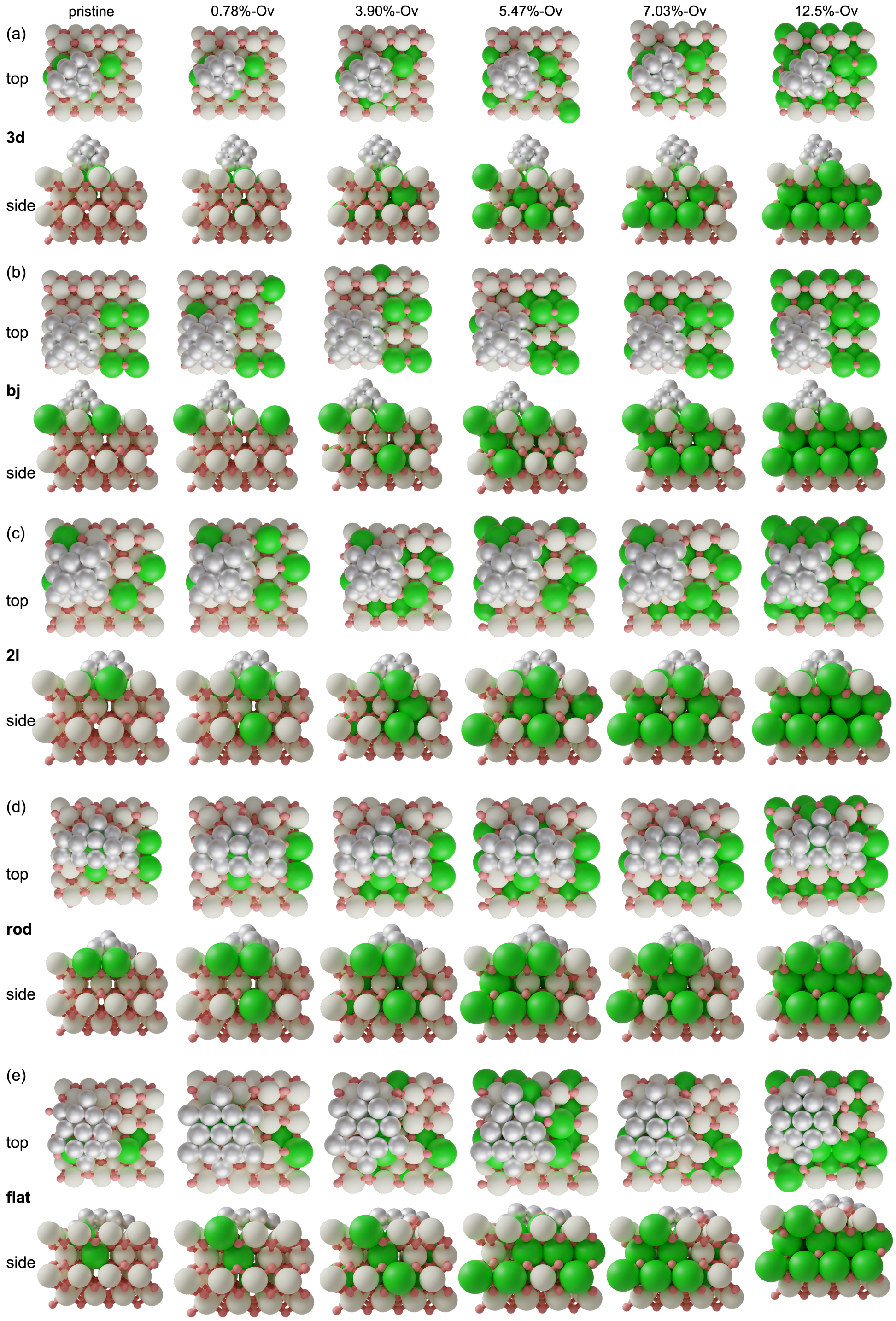

Figure S20: Representative optimized structures of Pt_{13}/CeO_{2-x} models with diverse O_v and polaron configurations. Each panel shows a unique sampling of O_v and Ce^{3+} (polaron) distributions. Top and side views are shown for each configuration. (a) Pt_{13}_3d/CeO_{2-x}. (b) Pt_{13}_bj/CeO_{2-x}. (c) Pt_{13}_2l/CeO_{2-x}. (d) Pt_{13}_rod/CeO_{2-x}. (e) Pt_{13}_flat/CeO_{2-x}.

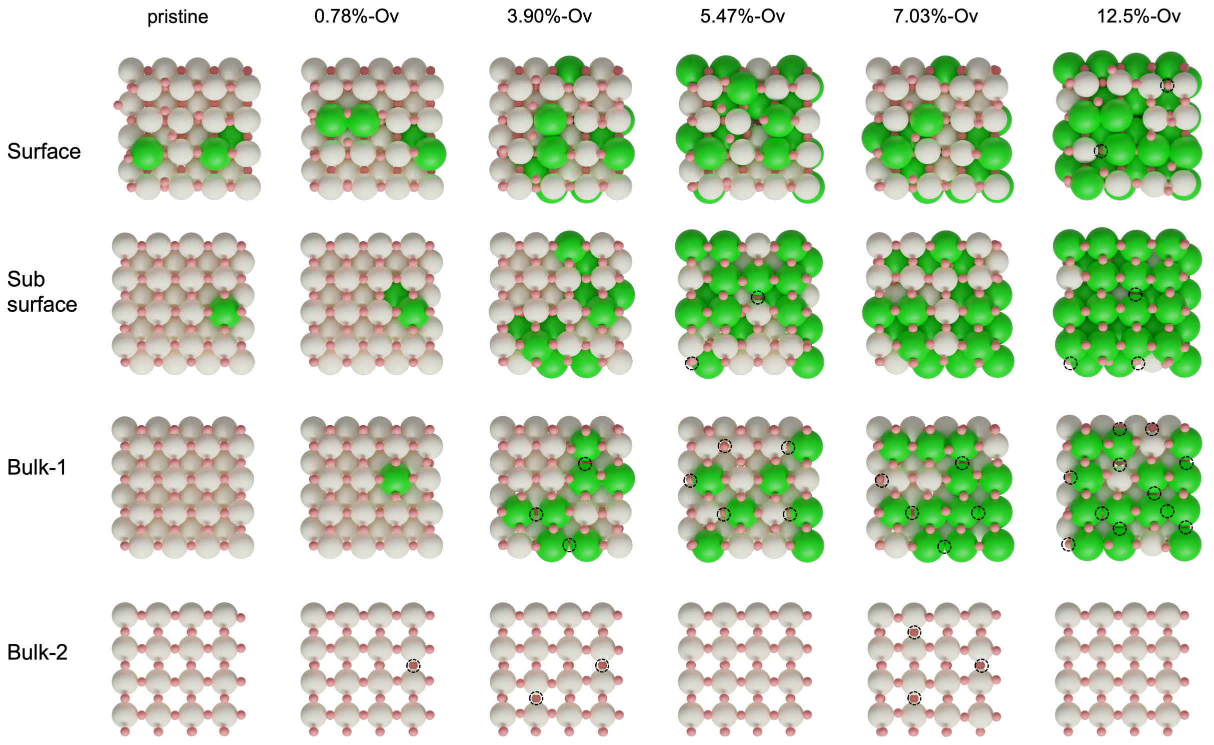

Figure S21: Layer-resolved visualization of oxygen vacancy positions and polaron distributions for the Pt_{13}_flat/CeO_{2-x} model at different oxygen vacancy concentrations. From top to bottom, the surface, subsurface, first bulk, and second bulk layers are shown separately. Oxygen vacancies are indicated by dashed circles, and Ce^{3+} sites associated with localized polarons are highlighted in green.

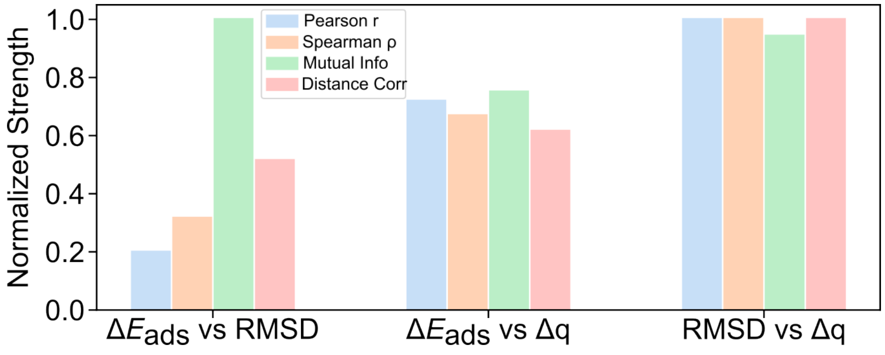

Figure S22: Normalized correlation strengths between the three pairs of features (ΔE_{ads} vs RMSD, ΔE_{ads} vs Δq, and RMSD vs Δq) calculated using different statistical methods (Pearson r, Spearman ρ, Mutual Information, and Distance Correlation).

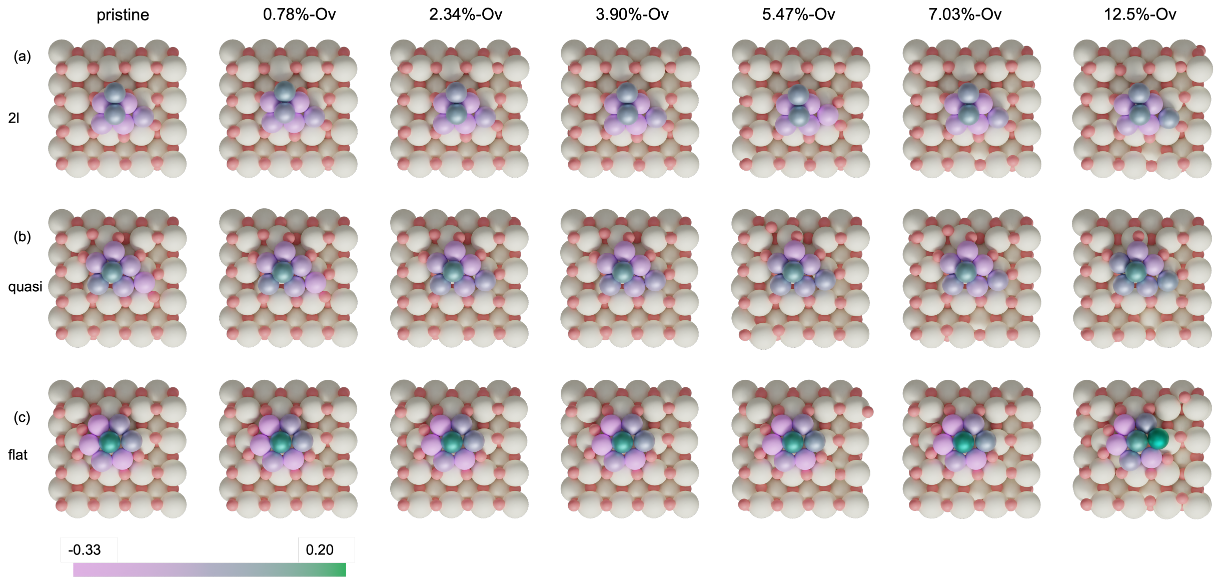

Figure S23: Charge distribution in Pt_7/CeO_{2-x}. (a) Pt_7_2l/CeO_{2-x}. (b) Pt_7_quasi/CeO_{2-x}. (c) Pt_7_flat/CeO_{2-x}. Each panel displays a unique atomic arrangement with atomic charges visualized by colour mapping (see colour bar, values in units of $|e^-|$). Pink colours correspond to Pt atoms with lower electron density (electron-deficient), while green colours indicate Pt atoms with higher electron density (electron-rich). White and red represent Ce and O atoms, respectively.

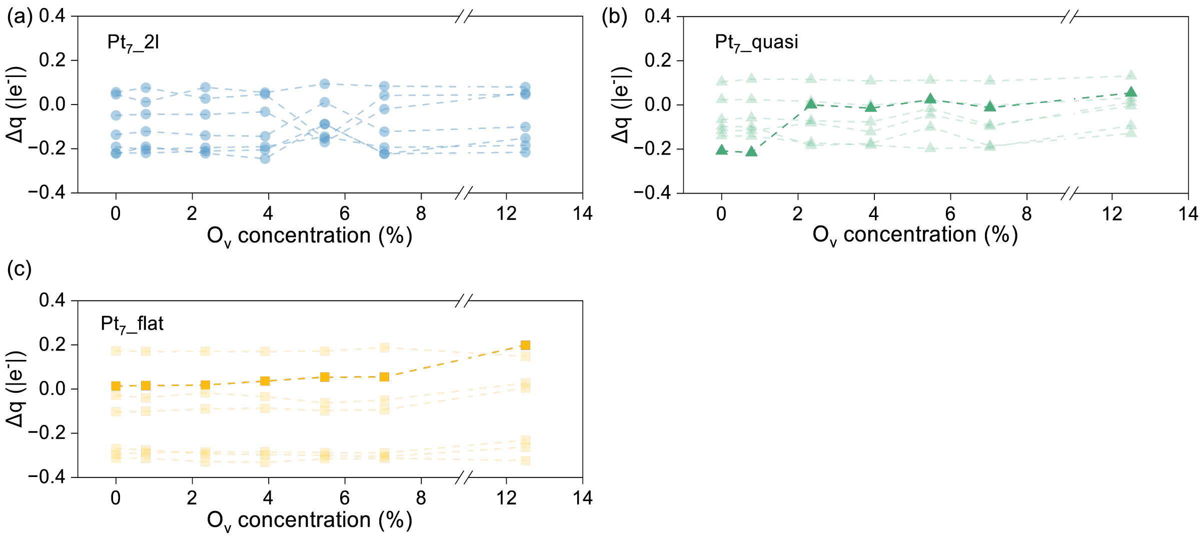

Figure S24: Charge variation (Δq) of individual Pt atoms in Pt_7/CeO_{2-x} as a function of O_v concentration. Positive Δq denotes electron gain by Pt from reduced CeO_{2-x} ($CeO_{2-x} \rightarrow Pt$), whereas negative Δq indicates electron donation from Pt to CeO_{2-x} ($Pt \rightarrow CeO_{2-x}$). Highlighted points mark configurations exhibiting a reversal in charge-transfer direction. The unit is $|e^-|$.

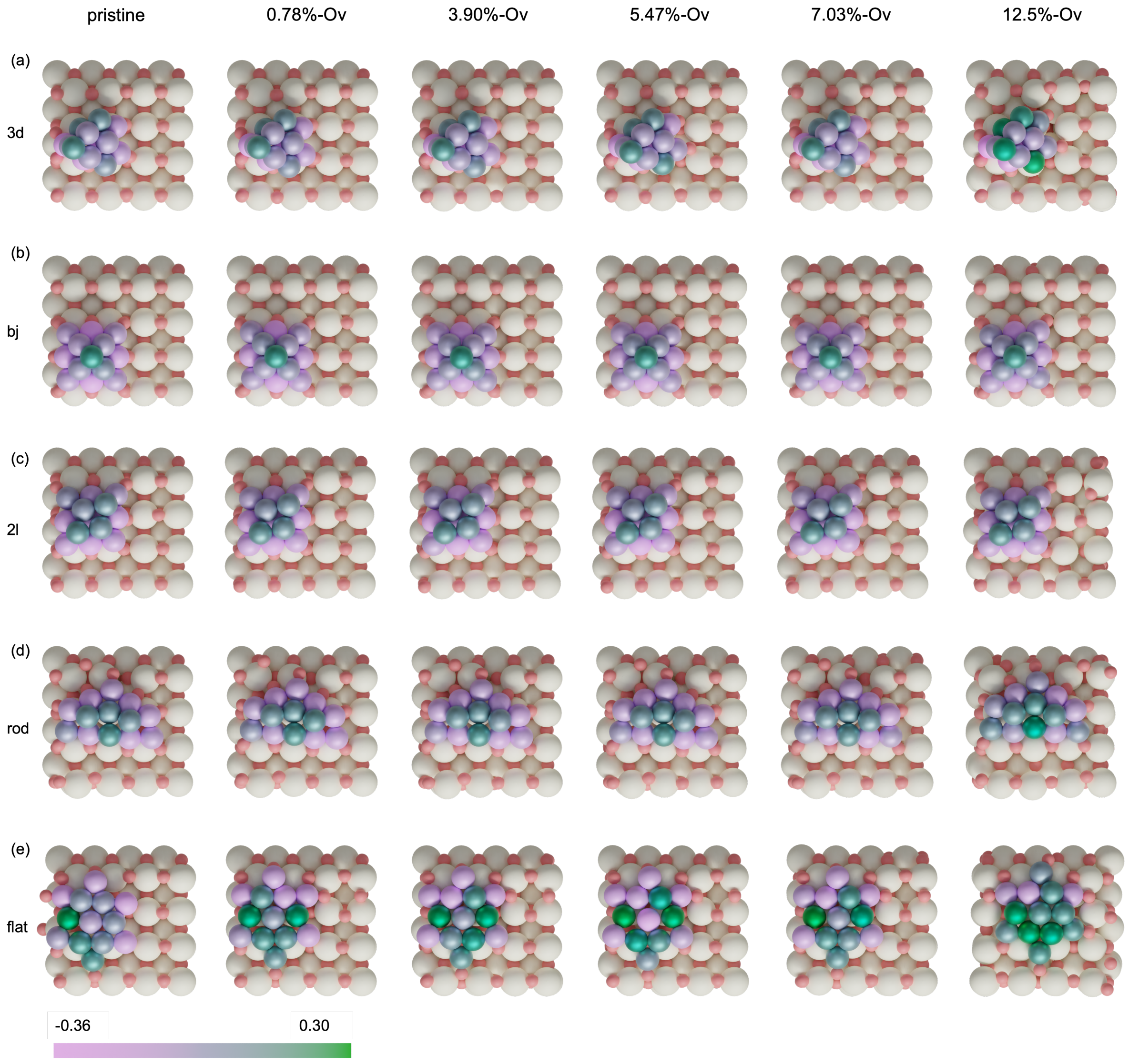

Figure S25: Charge distribution in Pt_{13}/CeO_{2-x}. (a) Pt_{13}_3d/CeO_{2-x}. (b) Pt_{13}_bj/CeO_{2-x}. (c) Pt_{13}_2l/CeO_{2-x}. (d) Pt_{13}_rod/CeO_{2-x}. (e) Pt_{13}_flat/CeO_{2-x}.

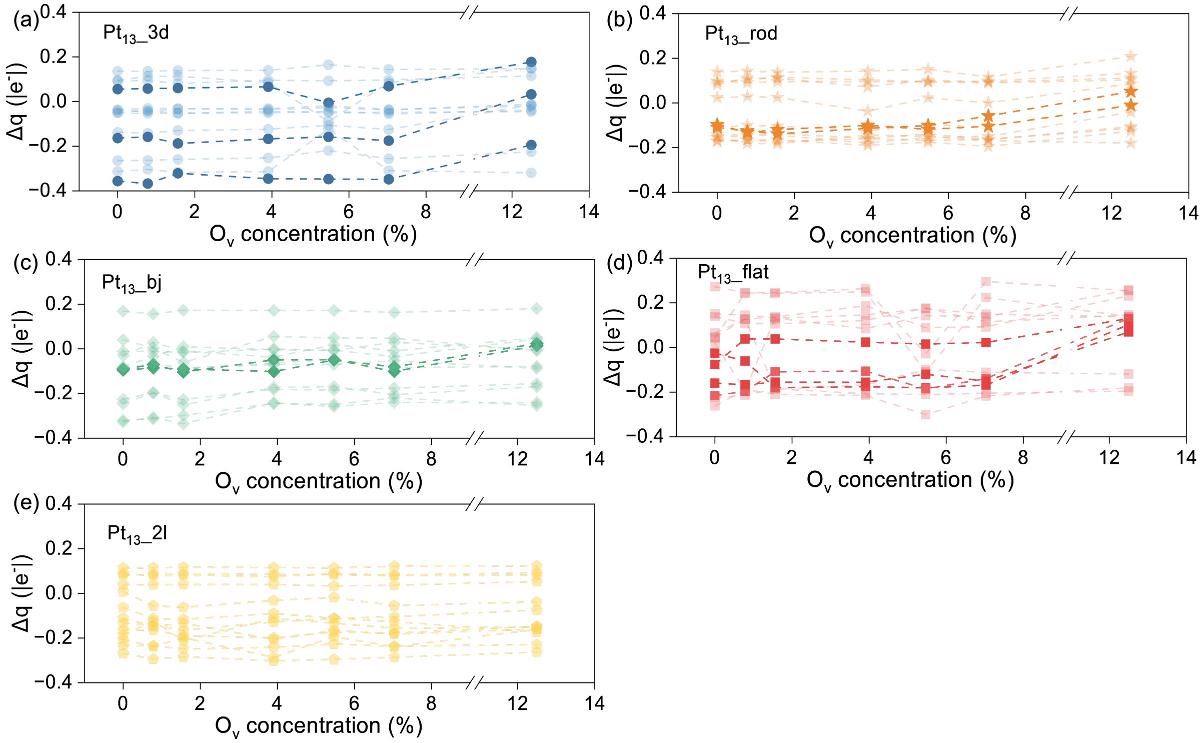

Figure S26: Charge variation (Δq) of individual Pt atoms in Pt_{13}/CeO_{2-x} as a function of O_v concentration. Highlighted points denote configurations exhibiting a reversal in charge-transfer direction.

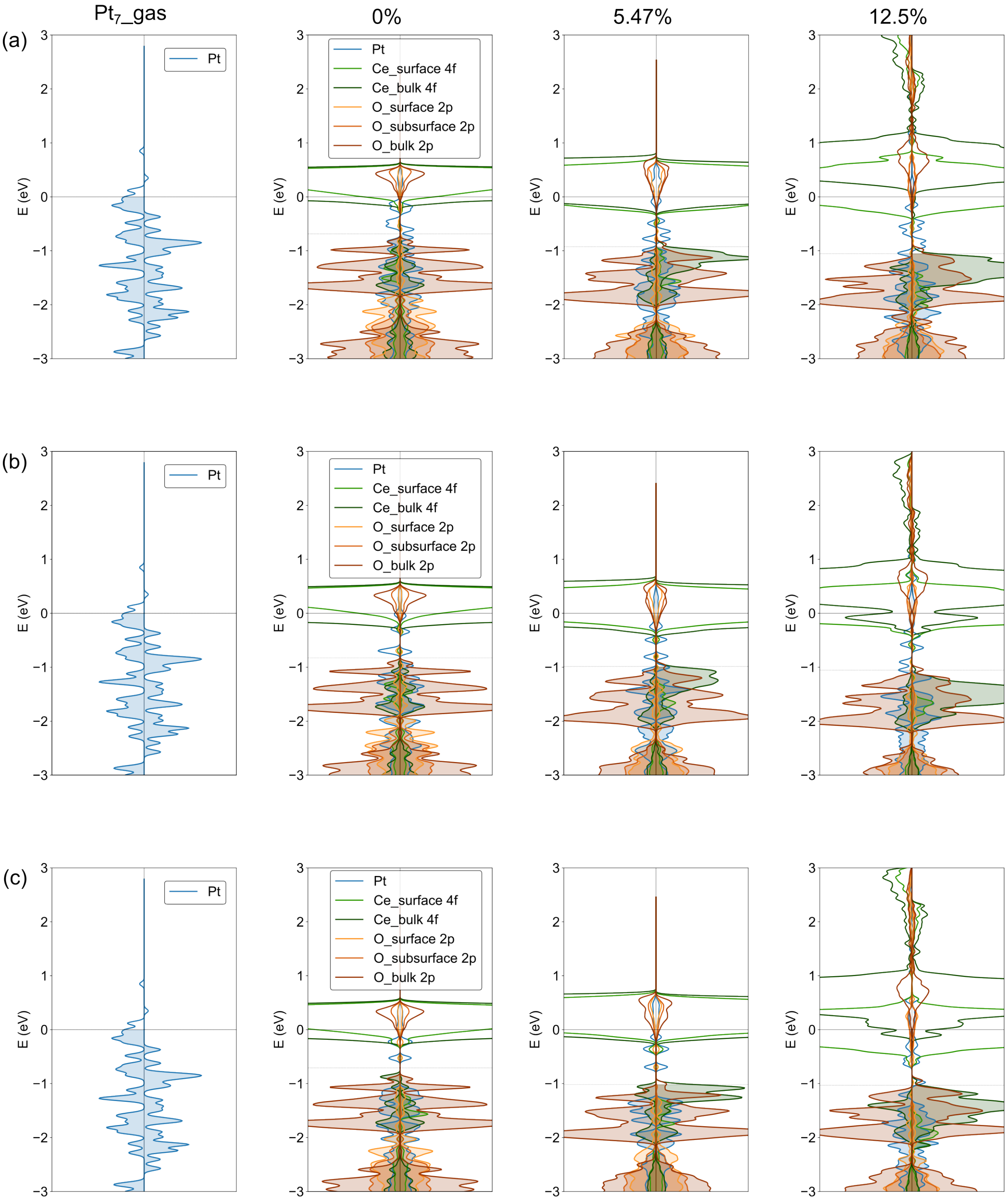

Figure S27: PDOS for representative Pt_7/CeO_{2-x} models, aligned by the O 2s band centre. (a) Pt_7_2l/CeO_{2-x}. (b) Pt_7_quasi/CeO_{2-x}. (c) Pt_7_flat/CeO_{2-x}. Each panel shows the element- and layer-resolved PDOS for Pt, Ce (surface/bulk 4f), and O (subsurface/bulk 2p) orbitals, as labelled. The energy zero ($E = 0$ eV).

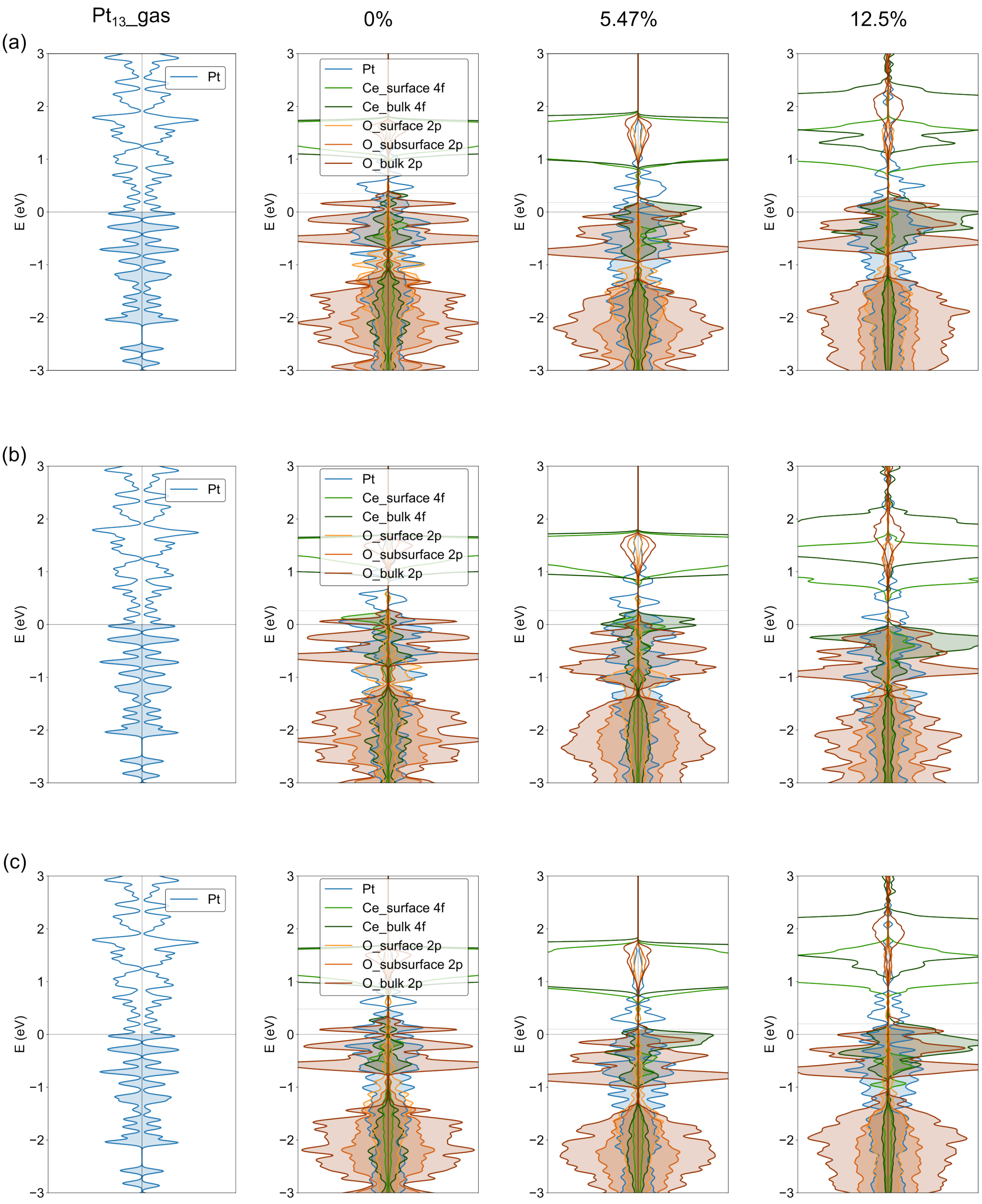

Figure S28: PDOS for representative Pt_{13}/CeO_{2-x} models, aligned by the O 2s band centre. (a) Pt_{13}_3d/CeO_{2-x}. (b) Pt_{13}_bj/CeO_{2-x}. (c) Pt_{13}_2l/CeO_{2-x}.

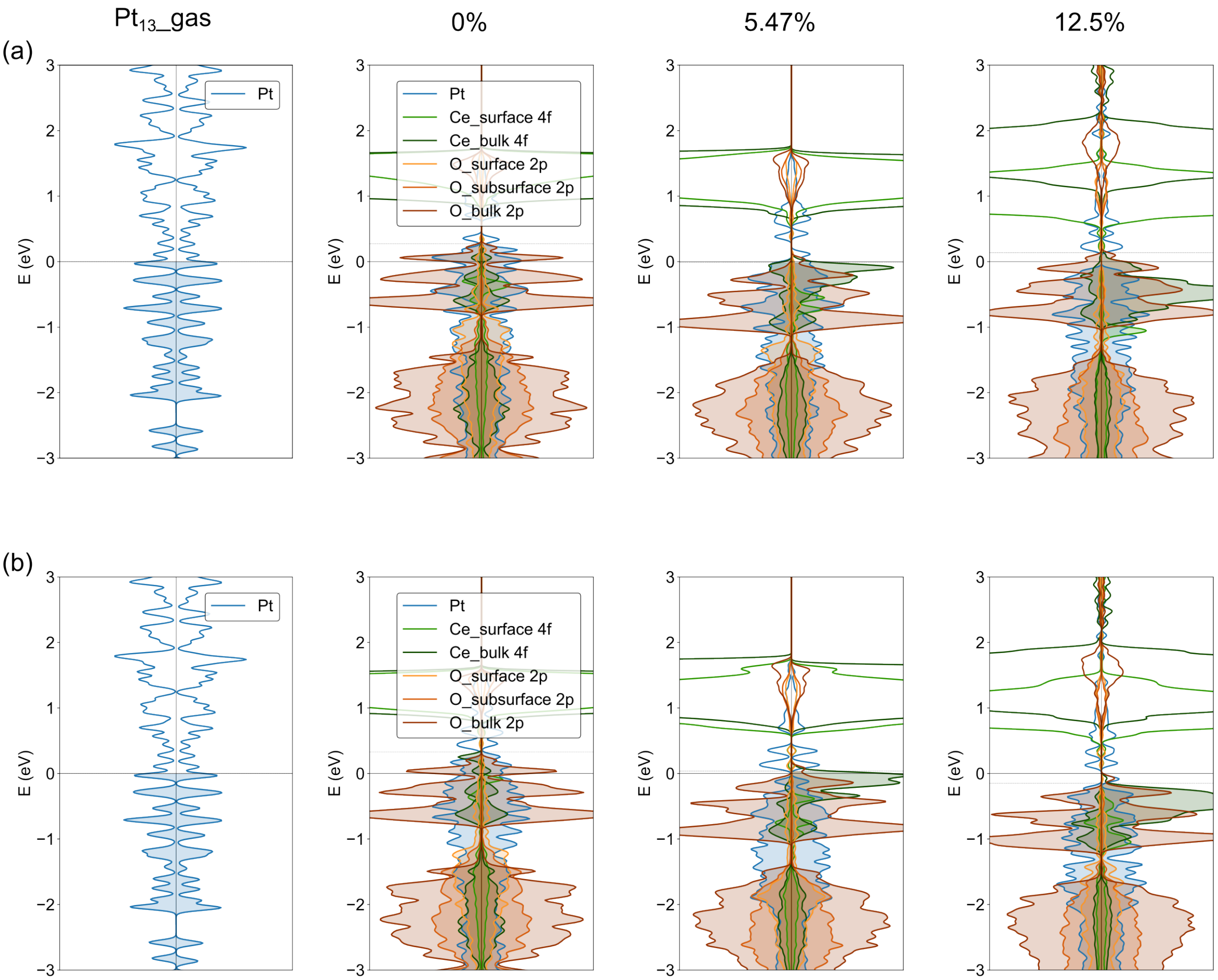

Figure S29: PDOS for representative Pt_{13}/CeO_{2-x} models, aligned by the O 2s band centre. (a) Pt_{13}_rod/CeO_{2-x}. (b) Pt_{13}_flat/CeO_{2-x}.

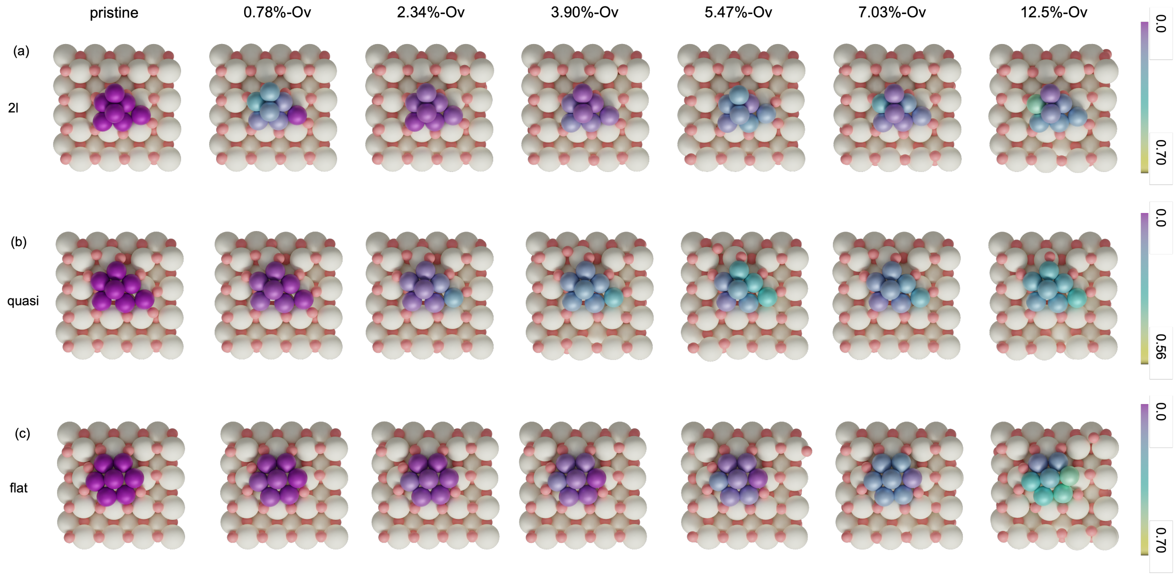

Figure S30: Local atomic RMSD of Pt_7/CeO_{2-x}. (a) Pt_7_2l/CeO_{2-x}. (b) Pt_7_quasi/CeO_{2-x}. (c) Pt_7_flat/CeO_{2-x}. Each panel shows a representative structure coloured by the local atomic RMSD, quantifying the deviation of each atom from its position in the corresponding pristine (defect-free) cluster of the same shape. RMSD values (in Å) are indicated by the colour bars. Purple to cyan colour mapping highlights the degree of local distortion within the Pt clusters across different sampled configurations.

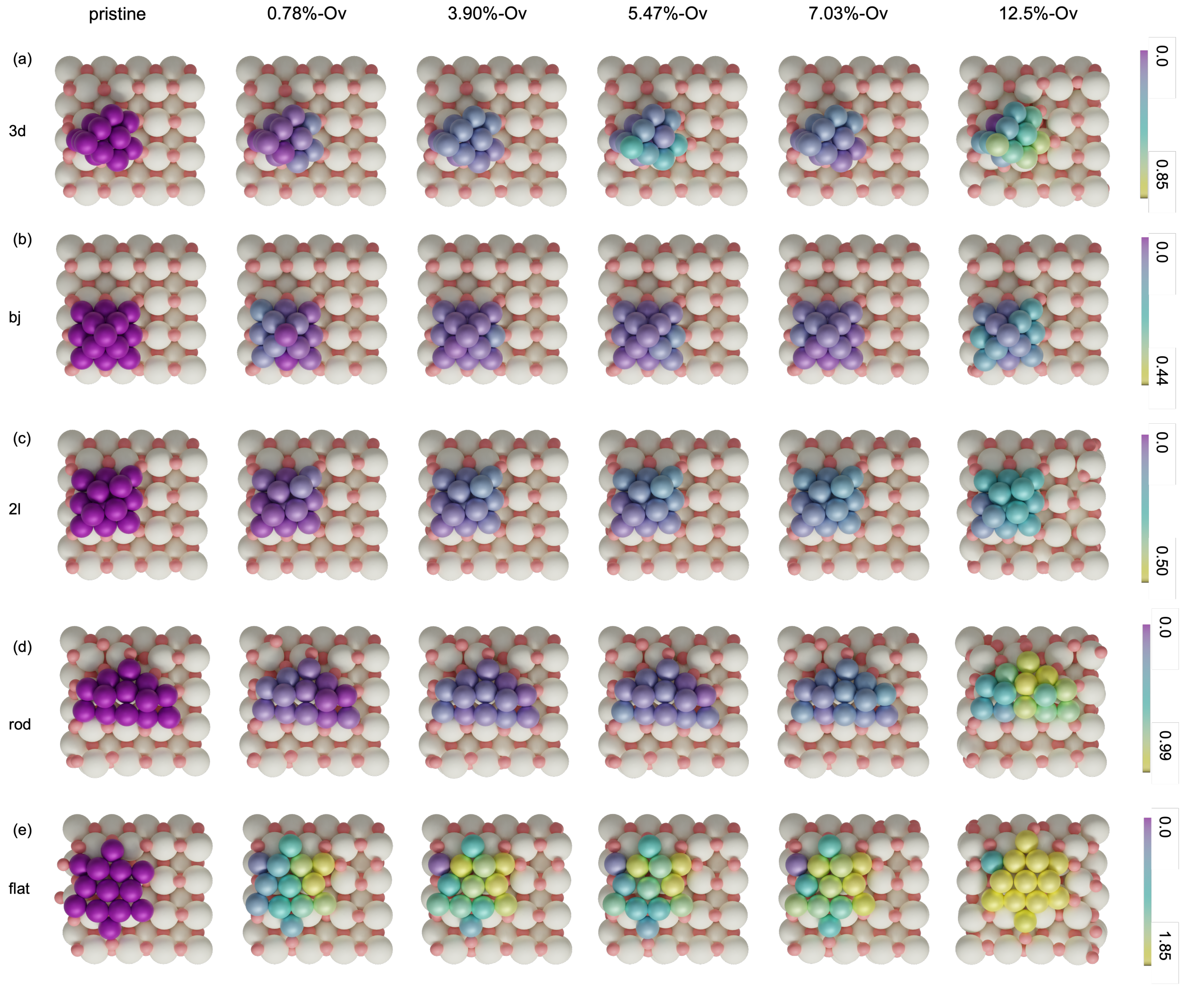

Figure S31: Local atomic RMSD of Pt_{13}/CeO_{2-x}. (a) Pt_{13}_3d/CeO_{2-x}. (b) Pt_{13}_bj/CeO_{2-x}. (c) Pt_{13}_2l/CeO_{2-x}. (d) Pt_{13}_rod/CeO_{2-x}. (e) Pt_{13}_flat/CeO_{2-x}.

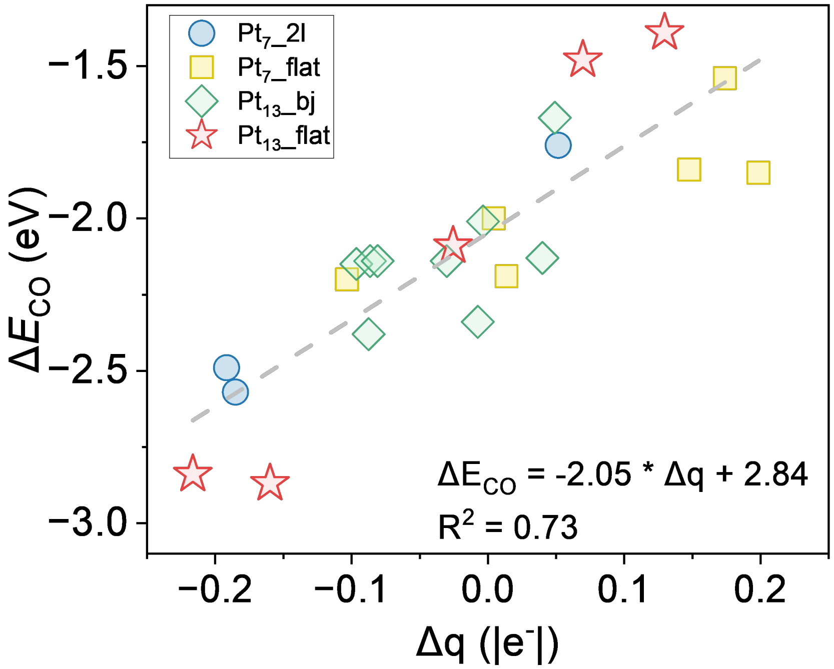

Figure S32: CO adsorption energy (ΔE_{CO}) plotted against the charge variation (Δq) of Pt atoms for representative Pt_7 and Pt_{13} configurations. Each point corresponds to a top-site CO adsorption on a specific Pt atom extracted from the optimized structures. Symbols denote different configurations: Pt_7_2l (blue circles), Pt_7_flat (yellow squares), Pt_{13}_bj (green diamonds), and Pt_{13}_flat (red stars).
A clear trend is observed in which electron-enriched Pt sites (positive Δq) exhibit weaker CO adsorption (less negative ΔE_{CO}). The linear fit yields an R^2 of 0.73, confirming the strong correlation between Δq and ΔE_{CO}. This demonstrates that the reversed charge transfer from CeO_{2-x} to Pt modulates CO binding strength and mitigates CO poisoning.

Table S2: Complete list of physicochemical features used for ML analysis. Features are grouped into Pt cluster, Pt–O interface, and CeO_{2-x} domains.

**Domain**	**Symbol**	**Description**	**Unit**
Pt cluster	Δq	Electron transfer from Pt cluster to CeO_{2-x} (Pt $\rightarrow$ CeO_{2-x}: negative)	$\lvert e^- \rvert$
	RMSD	Geometric distortion relative to gas-phase cluster	Å
Pt–O interface	Pt–O bond count	Total number of Pt–O bonds	–
	$\sum$(Pt–O dist)	Sum of all Pt–O bond lengths	Å
	Min(Pt–O dist)	Minimum Pt–O bond length	Å
	Mean(Pt–O dist)	Average Pt–O bond length	Å
	Max(Pt–O dist)	Maximum Pt–O bond length	Å
	Std(Pt–O dist)	Standard deviation of Pt–O bond lengths	Å
CeO_{2-x} support	O_v (%)	O_v concentration ((removed O / total O) $\times$ 100%)	%
	Surface polaron count	Number of surface-localized polarons	–
	Polaron count	Total number of polarons	–
	ε	Surface strain: average number of surface O atoms within 2.5 Å of Ce^{3+}	–
	$\sum$(Ce^{3+}–Ce^{3+} dist)	Total pairwise distance between Ce^{3+} sites	Å
	Min(Ce^{3+}–Ce^{3+} dist)	Minimum Ce^{3+}–Ce^{3+} distance	Å
	Mean(Ce^{3+}–Ce^{3+} dist)	Average Ce^{3+}–Ce^{3+} distance	Å
	Max(Ce^{3+}–Ce^{3+} dist)	Maximum Ce^{3+}–Ce^{3+} distance	Å
	Std(Ce^{3+}–Ce^{3+} dist)	Standard deviation of Ce^{3+}–Ce^{3+} distances	Å
	$E_{\text{pol-pol}}$	Polaron–polaron interaction energy ($1/\varepsilon_r$)	eV
	$E_{\text{pol-lattice}}$	Interaction energy of each Ce^{3+} with lattice centre (harmonic)	eV

Table S3: Main characteristics of the three machine-learning models

**Model**	**Learning strategy**	**Key characteristics**	**Main advantages**	**Main limitations**
GB	Sequential boosting	Each new tree corrects residuals from previous ones	Captures complex nonlinear relations; flexible	Sensitive to hyperparameters; slower training
RF	Parallel bagging	Builds many independent trees on random data subsets and averages their outputs	Robust; low variance; interpretable feature importance	May underfit highly nonlinear data
XGBoost	Regularized gradient boosting	Includes shrinkage, regularization, and column subsampling	High accuracy; efficient; prevents overfitting	Requires more parameter tuning; less interpretable

References

(1) Tian, W. Q. et al. Geometrical and electronic structure of the Pt_7 cluster: A density functional study. *The Journal of Physical Chemistry A* **2004**, *108*, 3806–3812.

(2) Mao, Z. et al. Ni Nanoparticles on CeO_2(111): Energetics, Electron Transfer, and Structure by Ni Adsorption Calorimetry, Spectroscopies, and Density Functional Theory. *ACS Catalysis* **2020**, *10*, 5101–5114.

(3) Kolsbjerg, E. L.; Peterson, A. A.; Hammer, B. Neural-network-enhanced evolutionary algorithm applied to supported metal nanoparticles. *Physical Review B* **2018**, *97*, 195424.

(4) Zhai, H.; Alexandrova, A. N. Local Fluxionality of Surface-Deposited Cluster Catalysts: The Case of Pt_7 on Al_2O_3. *The Journal of Physical Chemistry Letters* **2018**, *9*, 1696–1702.

(5) Capdevila-Cortada, M. et al. Reactivity descriptors for ceria in catalysis. *Applied Catalysis B: Environmental* **2016**, *197*, 299–312.

(6) Geiger, J.; López, N. Coupling Metal and Support Redox Terms in Single-Atom Catalysts. *The Journal of Physical Chemistry C* **2022**, *126*, 13698–13704.

(7) Pérez-Bailac, P.; Lustemberg, P. G.; Ganduglia-Pirovano, M. V. Facet-dependent stability of near-surface oxygen vacancies and excess charge localization at CeO_2 surfaces. *Journal of Physics: Condensed Matter* **2021**, *33*, 504003.

(8) Geiger, J. et al. Data-driven models for ground and excited states for Single Atoms on Ceria. *npj Computational Materials* **2022**, *8*, 1–8.

(9) Murgida, G. E. et al. Ordering of oxygen vacancies and excess charge localization in bulk ceria: A DFT + U study. *Physical Review B* **2014**, *90*, 115120.

(10) Tuller, H. L.; Nowick, A. S. Small polaron electron transport in reduced CeO_2 single crystals. *Journal of Physics and Chemistry of Solids* **1977**, *38*, 859–867.

(11) Birschitzky, V. C. et al. Machine learning for exploring small polaron configurational space. *npj Computational Materials* **2022**, *8*, 1–9.

(12) Pedregosa, F. et al. Scikit-learn: Machine Learning in Python. *Journal of Machine Learning Research* **2011**, *12*, 2825–2830.

(13) McKinney, W. Data Structures for Statistical Computing in Python. *scipy* **2010**,

(14) Hunter, J. D. Matplotlib: A 2D Graphics Environment. *Computing in Science & Engineering* **2007**, *9*, 90–95.

(15) Waskom, M. L. seaborn: statistical data visualization. *Journal of Open Source Software* **2021**, *6*, 3021.

(16) Virtanen, P. et al. SciPy 1.0: fundamental algorithms for scientific computing in Python. *Nature Methods* **2020**, *17*, 261–272.

(17) Snoek, J.; Larochelle, H.; Adams, R. P. Practical Bayesian Optimization of Machine Learning Algorithms. 2012; `http://arxiv.org/abs/1206.2944`.

(18) Friedman, J. H. Greedy Function Approximation: A Gradient Boosting Machine. *The Annals of Statistics* **2001**, *29*, 1189–1232.

(19) Breiman, L. Random Forests. *Machine Learning* **2001**, *45*, 5–32.

(20) Chen, T.; Guestrin, C. XGBoost: A Scalable Tree Boosting System. Proceedings of the 22nd ACM SIGKDD International Conference on Knowledge Discovery and Data Mining. San Francisco California USA, 2016; pp 785–794.

(21) Daelman, N.; Capdevila-Cortada, M.; López, N. Dynamic charge and oxidation state of Pt/CeO_2 single-atom catalysts. *Nature Materials* **2019**, *18*, 1215–1221.
